# Multifactorial Regulation of Myometrial Contractility During Pregnancy and Parturition

**DOI:** 10.3389/fendo.2019.00714

**Published:** 2019-10-25

**Authors:** Carole R. Mendelson, Lu Gao, Alina P. Montalbano

**Affiliations:** Departments of Biochemistry and Obstetrics and Gynecology, North Texas March of Dimes Birth Defects Center, The University of Texas Southwestern Medical Center, Dallas, TX, United States

**Keywords:** progesterone, gene regulation, transcription corepressor, inflammation, pregnancy, myometrium, NF-κB

## Abstract

The steroid hormones progesterone (P_4_) and estradiol-17β (E_2_), produced by the placenta in humans and the ovaries in rodents, serve crucial roles in the maintenance of pregnancy, and the initiation of parturition. Because of their critical importance for species survival, the mechanisms whereby P_4_ and its nuclear receptor (PR) maintain myometrial quiescence during pregnancy, and for the decline in P_4_/PR and increase in E_2_/estrogen receptor (ER) function leading to parturition, are multifaceted, cooperative, and redundant. These actions of P_4_/PR include: (1) PR interaction with proinflammatory transcription factors, nuclear factor κB (NF-κB), and activating protein 1 (AP-1) bound to promoters of proinflammatory and contractile/contraction-associated protein (CAP) genes and recruitment of corepressors to inhibit NF-κB and AP-1 activation of gene expression; (2) upregulation of inhibitors of proinflammatory transcription factor activation (IκBα, MKP-1); (3) induction of transcriptional repressors of CAP genes (e.g., ZEB1). In rodents and most other mammals, circulating maternal P_4_ levels remain elevated throughout most of pregnancy and decline precipitously near term. By contrast, in humans, circulating P_4_ levels and myometrial PR levels remain elevated throughout pregnancy and into labor. However, even in rodents, wherein P_4_ levels decline near term, P_4_ levels remain higher than the K_d_ for PR binding. Thus, parturition is initiated in all species by a series of molecular events that antagonize the P_4_/PR maintenance of uterine quiescence. These events include: direct interaction of inflammatory transcription factors (e.g., NF-κB, AP-1) with PR; increased expression of P_4_ metabolizing enzymes; increased expression of truncated/inhibitory PR isoforms; altered expression of PR coactivators and corepressors. This article will review various mechanisms whereby P_4_ acting through PR isoforms maintains myometrial quiescence during pregnancy as well as those that underlie the decline in PR function leading to labor. The roles of P_4_- and E_2_-regulated miRNAs in the regulation and integration of these mechanisms will also be considered.

## Introduction

Preterm birth (<37 weeks gestation), which affects ~15 × 10^6^ births globally each year, is a major cause of death within the first month of postnatal life (1). The highest rates of preterm birth (≥15% of all live births) occur in sub-Saharan Africa, Pakistan, and Indonesia. In the U.S., the preterm birth rate remains at ~10% of all overall live births. However, significant racial disparities in preterm birth rates exist, with the incidence of preterm birth among African-Americans being 50% higher than that of the overall population. Notably, the underlying causes for these racial differences remain unknown (2). Astonishingly, the modalities used to treat and/or prevent preterm labor have changed little over the past 50 years. This is due, in part, to our incomplete comprehension of mechanisms that mediate myometrial quiescence and contractility as well as the reluctance of pharmaceutical companies to engage in drug discovery in this critical area.

Throughout pregnancy, myometrial quiescence is controlled by increased progesterone (P_4_), secreted by the placenta and/or the ovarian corpus luteum, depending upon the species. In humans, two progesterone receptor (PR) isoforms, PR-A (94 kDa), and PR-B (114 kDa), alternative transcripts of a single gene (3, 4), mediate P_4_ action to block myometrial contractility. Both PR-A and PR-B bind to progesterone response elements (PREs) in DNA; however, PR-A contains two of three transcriptional activation domains that are present in PR-B and is, therefore, less transcriptionally active. Thus, PR-A can repress PR-B transcriptional activity in a cell- and gene-specific context (5, 6). PR-A was also found to inhibit PR-B transcriptional activity in cultured human myometrial cells (7), suggesting a potential antagonistic role of PR-A on PR-B action in the myometrium. PR-A and PR-B are differentially regulated in the human myometrium during pregnancy (8); the ratio of PR-A to PR-B mRNA (9) and protein (7) was observed to increase significantly in the myometrium of women in labor when compared to those not in labor at term. In telomerase-immortalized human myometrial (hTERT-HM) cells stably expressing either PR-A or PR-B, P_4_ treatment had increased anti-inflammatory activity in PR-B-expressing cells when compared to those expressing PR-A (10).

As described below, a number of unique and redundant mechanisms mediate the action of P_4_/PR to maintain uterine quiescence. In rodents and most other mammals, circulating maternal P_4_ levels remain elevated throughout most of pregnancy and decline sharply prior to parturition (11). This has led to the concept that labor is associated with P_4_ withdrawal. By contrast, in humans and guinea pigs circulating P_4_ and myometrial levels of PR fail to decline during late pregnancy and into labor (12). However, treatment with PR antagonists can cause increased myometrial contractility, cervical ripening, and/or increased sensitivity to labor induction by contractile factors (13–16). Importantly, even in rodents, circulating maternal P_4_ levels at term remain well above the equilibrium dissociation constant for binding to PR (17). Moreover, in rodents, local metabolism of P_4_ within the cervix and myometrium to inactive products near term is essential for the normal timing of parturition. Thus, in mice deficient in 5α-reductase type I (expressed in cervix) (18, 19) or 20α-hydroxysteroid dehydrogenase (20α-HSD, expressed in myometrium) (20) parturition is severely delayed. Collectively, these findings have led to the concept that parturition in all placental mammals is initiated by a conserved sequence of molecular events that impairs the capacity of the PR to maintain uterine quiescence. These include: (1) direct interaction of transcription factor nuclear factor κB (NF-κB) with PR; (2) upregulation of P_4_ metabolizing enzymes within the uterus and cervix; (3) increased expression of truncated/inhibitory PR isoforms; (4) altered expression of key PR-interacting coactivators and corepressors. This article will review the various mechanisms whereby P_4_ acting through PR isoforms maintains myometrial quiescence during pregnancy as well as those that underlie the decline in PR function leading to parturition.

## Parturition is Associated With an Increased Inflammatory Response

**Figure 1:**
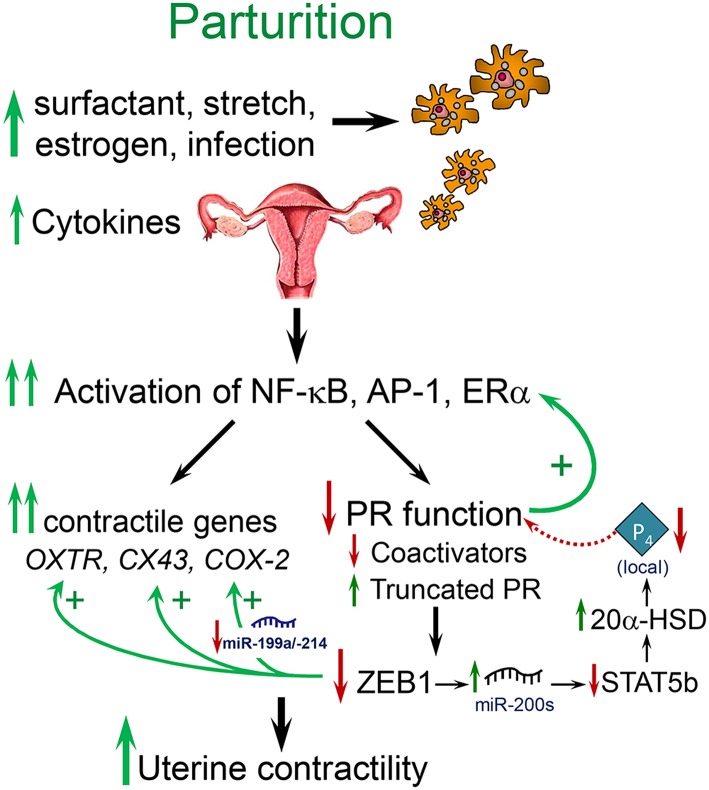
Labor at term or preterm is associated with an inflammatory response. Near term, signals from the fetus (e.g., increased surfactant lipoprotein secretion, increased placental CRH, cortisol, and estrogen) and mother (e.g., uterine stretch with increased chemokine production, increased estrogen receptor signaling), cause increased immune cell invasion of the fetal membranes, decidua, and myometrium. At preterm, immune cell invasion is stimulated by ascending infection associated with chorioamnionitis as well as increased uterine stretch in twin or multi-fetal pregnancies. The invading immune cells produce proinflammatory cytokines that cause activation of inflammatory transcription factors, such as NF-κB and AP-1, which promote activation of proinflammatory and *CAP* gene expression and a decline in PR function, caused by a decrease in coactivators and increased expression of PR-A and other truncated PR isoforms. The decline in PR function results in decreased ZEB1 expression, with increased expression of contractile genes (*OXTR* and *CX43*) and miR-200 family members and decreased expression of the miR-199a/214 cluster. The increase in miR-200s suppress their targets ZEB1/2 and STAT5b and increase local P_4_ metabolism via upregulation of 20α-HSD. The decrease in miR-199a/214 results in upregulation of their target, COX-2, and an increase in synthesis of contractile prostaglandins. These events culminate in an increase in myometrial contractility leading to parturition. Modified from Mendelson et al. (25).

Term and preterm parturition is initiated by an enhanced inflammatory response, increased levels of proinflammatory cytokines in amniotic fluid (21) and the invasion of the fetal membranes, cervix and myometrium by neutrophils and macrophages (Mϕ) (22–24) (Figure 1). The secretion of cytokines and chemokines by the invading immune cells (26) cause activation of NF-κB and other inflammation-associated transcription factors (e.g., AP-1) (23, 27–30). These activated transcription factors promote increased expression of myometrial proinflammatory [e.g., interleukin (*IL*)*-1*β*, IL-8*] and contractile/CAP [connexin-43 (*CX43/GJA1*), oxytocin receptor (*OXTR*), and cyclooxygenase 2 (*COX-2/PTGS2*)] genes, leading to parturition (31–34). Whereas, intra-amniotic infection associated with chorioamnionitis can provide the stimulus for the inflammatory response leading to preterm labor (35), signals from both mother and fetus provide critical inflammatory stimuli leading to labor at term.

### Increased Mechanical Stretch of the Uterus Results in Production of Chemokines, Enhanced Immune Cell Invasion, and Proinflammatory Signaling Leading to Labor

Near term, enhanced uterine stretch caused by the growing fetus provides an important stimulus for the initiation of labor (36, 37) (Figure 1). The increased incidence of preterm birth in twin and multiple, as compared to singleton, pregnancies implicates uterine over-distension as a causative factor (38). The expression of the β-chemokine, monocyte chemoattractant protein-1/C-C motif ligand 2 (MCP-1/CCL2), which attracts and activates Mϕ, was found to be upregulated in the term pregnant myometrium of women in labor, as compared to myometrium from women not in labor (39) and of pregnant rats prior to and during parturition (36). In pregnant rats carrying pups only in one uterine horn, increased MCP-1 expression was observed only in the gravid horn, implying the potential role of the fetus and/or of uterine stretch (36) in the induction of MCP-1 expression. Furthermore, the findings that MCP1 expression and Mϕ infiltration were greatly increased in the pregnant rat uterus with preterm labor induction by the PR antagonist, mifepristone/RU486, and inhibited by progestin treatment to delay parturition (36) suggests a role of P_4_/PR in MCP-1 regulation. Preterm labor was induced in non-human primates by intrauterine balloon inflation, in association with increased expression of IL-6, IL-8, and CCL-2 in the myometrium (40). Thus, enhanced inflammation associated with mechanical stress contributes to the initiation of term and preterm labor. In studies using human myometrial smooth muscle cells in culture, IL-1β and TNFα induced expression of MCP-1 and other chemokines; this was blocked by an inhibitor of the NF-κB signaling pathway (41). Likewise, in human choriodecidual and breast cancer cells, MCP-1 was stimulated by NF-κB activation and inhibited by P_4_/PR (42).

### Increased Estrogen Receptor Signaling Contributes to the Inflammatory Response Leading to Parturition

Across mammalian species, an increase in circulating estradiol-17β (E_2_) (43, 44) and/or myometrial estrogen receptor α (ERα) activity (9, 45) precedes the increase in uterine contractility near term (Figure 1). Estrogens induce migration of immune cells to the uterus and antagonize anti-inflammatory actions of P_4_/PR (9, 46). Moreover, ERα activation enhances transcription of the *CAP* genes, *OXTR* (47), *CX43* (48), and *COX-2* (9), and the resulting synthesis of prostaglandins that increase myometrial contractility (49–51). These actions of estrogen may be mediated, in part, through interaction of ERα and p160 coactivators with the AP-1 transcription factors Fos and Jun at AP-1-regulated promoters, resulting in an increase in AP-1 transcriptional activity (52).

Interestingly, we observed that ERα is a direct target of the microRNA, miR-181a, which significantly declines in mouse myometrium near term and in term myometrial tissues from women in labor, compared to those not-in-labor (53). Furthermore, E_2_ treatment inhibited miR-181a expression in uteri of ovariectomized mice and in human myometrial cells in primary culture. This revealed the presence of a feedback loop, wherein increased circulating E_2_ near term causes suppression of miR-181a, resulting in upregulation of ERα with further downregulation of miR-181a (53). In human myometrial cells, overexpression of miR-181a mimics repressed TNFα, CCL-2 and CCL-8 expression, while expression of the anti-inflammatory cytokine, IL-10, increased (53). TNFα was confirmed as a direct target of miR-181a, while CCL-2 and CCL-8 are predicted targets of this miRNA (53). c-Fos, which increases in pregnant rat (54) and mouse (53) myometrium during late gestation and into labor, was validated as a target of miR-181a in dendritic cells (55). These collective findings suggest that, from early through mid-gestation, relatively low E_2_/ERα levels allow increased expression miR-181a in myometrium, which represses ERα, c-FOS, TNFα, and several other proinflammatory cytokines, and increases the expression of anti-inflammatory cytokines. Moreover, near term increased circulating levels of E_2_ inhibit miR-181a, which allows the upregulation of its targets, ERα, TNFα, other proinflammatory cytokines, and transcription factor, c-FOS. In turn, c-FOS mediates the proinflammatory effects of E_2_/ERα and cytokines, which activate *CAP* genes and lead to labor.

**Figure 2:**
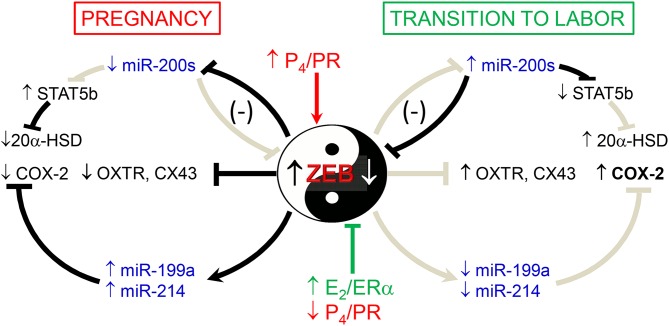
Opposing actions of P_4_ and E_2_ on myometrial contractility during pregnancy and labor are mediated by ZEB1 and ZEB2 and miRNAs. During pregnancy, increased P_4_/PR function causes the induction of ZEB1, which in turn inhibits expression of the miR-200 family and *CAP* genes and enhances expression of miR-199a-3p and miR-214, which cause suppression of their target, COX-2. Decreased levels of miR-200 family members cause a further increase in ZEB1 and enhance ZEB2 as well as STAT5b, which suppresses 20α-HSD expression to maintain increased tissue levels of P_4_. During the transition to labor, the decrease in P_4_/PR and increase in E_2_/ER function cause a decline in ZEB1. This allows for the upregulation of miR-200 family expression, causing further suppression of ZEB1 and inhibition of ZEB2 as well as STAT5b. The decrease in STAT5b allows upregulation of 20α-HSD and increased local metabolism of P_4_ to inactive products, further reducing PR function. The decline in ZEB1/2 allows upregulation of *CAP* genes and causes a decrease in *miR-199a/-214* expression, which allows for the induction of COX-2 and production of contractile prostaglandins. Collectively, these events culminate in parturition. Reproduced from Renthal et al. (58).

We also previously observed that in concert with the increased expression of the miR-200 family in pregnant mouse myometrium between 15.5 days post-coitum (dpc) and term (18.5 dpc and in labor) (56), there was a decline in the expression of the miR-199a/miR-214 cluster of miRNAs (57) (Figure 2). This was mediated by increased E_2_/ERα and the decrease in PR function, which inhibited expression of transcription factor ZEB1, a positive regulator of *miR-199a/miR-214* transcription (57, 58). Of note, miR-199a-3p and miR-214 directly target COX-2, which increases in the myometrium near term and during labor. Therefore, stimulatory effects of E_2_ on COX-2 expression (50) are likely mediated, in part, by its inhibition of miR-199a-3p/miR-214. Since miR-181a targets both ERα and cFOS (53), we suggest that the coordinate decline in miR-181a and miR-199a-3p/214 in the myometrium toward term mediates the induction of COX-2 expression via indirect and direct mechanisms.

### The Fetus Produces Signaling Molecules for the Initiation of Parturition

The fetus has been proposed to contribute to the initiation of parturition through the production of signaling molecules from its adrenals, placenta and lungs.

#### Cortisol Production by the Fetal Adrenal

In sheep, increased cortisol production by the fetal adrenal has been implicated in the initiation of parturition via the activation of placental COX-2 and production of prostaglandins (59). The prostaglandins, in turn, stimulate 17α-hydroxylase/17,20 lyase (CYP17) expression, resulting in increased placental production of C_19_-steroids, which are metabolized to estrogens by placental aromatase P450 (CYP19). The increased estrogens may antagonize PR function (12) and upregulate *CAP* gene expression via the mechanisms described above. The surge of fetal cortisol also promotes fetal lung maturation and synthesis of surfactant components (60), which, as described below, also serve as fetal signals for the initiation of labor.

#### Corticotropin-Releasing Hormone (CRH)

In humans, the placenta lacks the capacity to express *CYP17* and produce C_19_-steroids, which are instead synthesized in large quantities by the fetal adrenals (61). However, the human placenta is unique in its ability to secrete CRH (62), which is produced in increasing amounts near term and has been suggested to provide a fetal signal for the initiation of parturition (63, 64). Fetal placental CRH is proposed to upregulate secretion of adrenocorticotropic hormone (ACTH) by the fetal pituitary, which stimulates production of cortisol and the C_19_-steroid, dehydroepiandrosterone sulfate (DHEAS), by the fetal adrenals. DHEAS is subsequently metabolized within the placenta to estrogens, which, as mentioned, mediate inflammatory signaling leading to labor. CRH mRNA is also expressed at high levels in the bronchiolar epithelium of 13.5–17.5 dpc fetal mouse lung (65). It has been suggested that CRH may act directly within the fetal lung to promote synthesis of the major surfactant protein, surfactant protein (SP)-A. Accordingly, in *CRH* null mouse fetuses of *CRH*-deficient mothers, lung maturation and induction of *SP-A* expression were found to be delayed (66). Thus, CRH may act directly within the fetal lung to stimulate the production of surfactant components, and/or to increase fetal ACTH and adrenal cortisol production to enhance fetal lung development and surfactant synthesis. Accordingly, as described below, augmented surfactant production by the maturing fetal lung likely serves as an important fetal signal for the initiation of labor.

#### Surfactant Components Secreted by the Fetal Lung

Increased production of pulmonary surfactant components by the maturing fetal lung is proposed to signal the initiation of parturition (23, 67–70). Lung surfactant is a glycerophospholipid-rich surface-active lipoprotein produced specifically by type II cells of the pulmonary alveoli, which acts to reduce surface tension at the alveolar air-liquid interface after birth. Surfactant synthesis by the developing lung is initiated after ~85% of gestation is complete. Consequently, premature infants born prior to this time are at risk of developing respiratory distress syndrome due to surfactant deficiency. Dipalmitoylphosphatidylcholine (DPPC) is the major surfactant glycerophospholipid and most surface-active component. Approximately 10% of surfactant composition is comprised of the essentially lung-specific proteins SP-A, SP-B, SP-C, and SP-D (71, 72). SP-B and SP-C are lipophilic peptides produced from larger precursors. SP-B serves an essential role, together with DPPC, in the reduction of alveolar surface tension (71, 73). SP-A and SP-D serve as C-type lectin components of the innate immune system (74) that enhance the uptake of a variety of microbes by Mϕ (74–77).

The role of surfactant components in the initiation of parturition was first suggested by the finding that surfactant isolated from human amniotic fluid stimulated prostaglandin synthesis in amnion discs (67). It was proposed that amniotic fluid surfactant phospholipids provide a source of arachidonic acid as a substrate for synthesis of contractile prostaglandins. Others suggested that a substance in human amniotic fluid secreted in urine from the fetal kidney enhanced PGE_2_ production by human amnion cells (78); however, this amniotic fluid “substance” is likely derived from the fetal lung. Accordingly, Johnston and colleagues (79) suggested that platelet-activating factor (PAF), a potent proinflammatory phospholipid secreted into amniotic fluid with fetal lung surfactant near term, may enhance myometrial contractility leading to labor. Our laboratory has obtained extensive evidence for the roles of fetal lung SP-A and PAF as key fetus-derived inflammatory signals for the initiation of parturition (23, 69, 70).

##### SP-A and Toll-like receptor 2 (TLR2)

In all species studied, synthesis of SP-A by the fetal lung is developmentally upregulated with surfactant glycerophospholipids after ~85% of gestation is complete (80). Consequently, SP-A serves as a relevant marker of fetal lung maturity and surfactant production. SP-A expression in mouse fetal lung and its secretion into amniotic fluid are upregulated at 17.5 dpc and continue to increase toward term (19.5 dpc) (23, 81, 82). This is temporally associated with increased proinflammatory cytokine production by amniotic fluid Mϕ, their migration to the maternal uterus and the activation of uterine NF-κB (23). In studies using *Rosa 26 Lac-Z* mice, we observed that fetal Mϕ migrated to the maternal uterus with the induction in SP-A expression by the fetal lung during late gestation. Moreover, an intra-amniotic injection of SP-A caused preterm delivery of fetuses, and was associated with the activation of uterine NF-κB within 4.5 h. Conversely, injection of an SP-A antibody or NF-κB inhibitor into amniotic fluid delayed labor by >24 h (23). These findings suggested that enhanced SP-A secretion by the fetal lung near term causes activation and migration of fetal AF Mϕ to the maternal uterus, where increased cytokine production activates NF-κB and a signaling cascade, leading to labor. It should be noted that studies using laser capture of limited numbers of CD68^+^ or CD14^+^ (Mϕ markers) cells from the superficial portion of the myometrium from women carrying a male fetus at term failed to identify fetal mononuclear cells (83). Moreover, incubation of human amnion disks with SP-A resulted in upregulation of anti-inflammatory cytokines and cytokine receptors (84). However, SP-A can have both pro- and anti-inflammatory actions depending upon the cellular environment, as well as the receptor to which it binds (85).

To further study the roles of SP-A, the related C-type lectin, SP-D, and their putative receptor, TLR2 (86–89), in the initiation of parturition, we utilized gene-targeted mice. In first pregnancies, *SP-A*^−/−^ and *SP-A*^−/−^*/SP-D*^−/−^ female mice bred to genetically like males delivered at term (19.5 dpc). However, in subsequent pregnancies, these gene-targeted mice manifested a ~12 h delay in parturition, associated with significantly reduced levels of myometrial Cx43, Oxtr, IL-1β, and IL-6 mRNA at 18.5 dpc compared to wild-type (WT) mice (69). We postulated that the parturition timing difference in the deficient mice in first vs. second pregnancies was due to the dominant role of uterine mechanical stretch as a signal for parturition (36, 37, 90) in first pregnancies. However, in subsequent pregnancies, prior adaptation of the uterus to stretch (91) may allow other signals (e.g., surfactant proteins) to play a more significant role. *TLR2*^−/−^ females manifested a significant delay (~12 h) in parturition timing during first pregnancies, as well as reduced expression of *CAP* genes and the Mϕ marker, F4/80, in myometrium at term compared to WT (69). F4/80^+^ AF Mϕs from *TLR2*^−/−^ and *SP-A/D*^−/−^ mice expressed significantly lower levels of both pro-inflammatory and anti-inflammatory activation markers, compared to those of gestation-matched WT mice (69). These findings suggested that SP-A and SP-D act via TLR2 on fetal-derived Mϕ to modulate parturition timing; their impact may depend upon parity.

##### Roles of SRC-1 and SRC-2 in production of fetal signals leading to labor

Previously, we observed that the p160 family members (92), steroid receptor coactivators, SRC-1, and SRC-2/TIF-2, are critical for transcriptional upregulation of *SP-A* gene expression in fetal lung type II cells (93–95). SRCs do not bind to DNA directly; however they regulate gene transcription by interacting with steroid receptors and other transcription factors and by recruiting other coregulators with histone-modifying activities (92, 96) to alter chromatin structure (97). Notably, gene-targeted mice that are singly-deficient in *Src-1, Src-2*, or *Src-3* manifest various reproductive phenotypes (96). Importantly, mice that were double-knockout (dKO) for *Src-1* and *Src-2* died at birth from respiratory distress (98), which is indicative of lung surfactant deficiency. This observation was of great interest to us, considering the critical roles of SRC-1 (94) and SRC-2 (93) in *SP-A* expression.

To characterize these mice further, we crossed *Src-1*^+/−^/*Src-2*^+/−^ (*Src-1/-2 dhet*) males and females. Remarkably, they manifested severely delayed parturition (~38 h). This parturition delay occurred with significant reductions in NF-κB activation, as well as decreased expression of *Oxtr, Cx43, PGF*_2_α *synthase/Akr1b3* and levels of the contractile prostaglandin, PGF_2_α, in the maternal myometrium. The decrease in myometrial PGF_2_α was associated with impaired luteolysis and elevated circulating P_4_ (70). Notably, parturition timing was normal in *Src-1-KO* females and in *Src-2*^+/−^ females bred to genetically like males, revealing that *Src-1*/*-2* double-deficiency is requisite for the delay in parturition. Importantly, WT females bred to *Src-1*/-*2* double-deficient males exhibited a parturition delay equivalent to that observed in our crosses of *Src-1/-2* double-deficient males and females, with decreased myometrial NF-κB activation and *CAP* gene expression and elevated circulating P_4_. These findings indicated that the defect responsible for delayed parturition with *Src-1/-2* double deficiency was fetal in origin. Because of the importance of fetal lung SP-A production in the timing of parturition, it was of great interest that SP-A levels were significantly reduced in the lungs and amniotic fluid of fetuses doubly deficient in Src-1 and Src-2 when compared to WT. On the other hand, levels of SP-A in lungs and amniotic fluid of Src-1 or Src-2 singly deficient fetuses were similar to WT.

Clearly, the increase in gestation length in mice carrying *Src-1/-2* doubly deficient fetuses (~38 h) was significantly greater than what we observed in *SP-A-*deficient and in *SP-A/SP-D* double-deficient mice (~12 h). This suggested that signaling molecules, other than SP-A and SP-D, were affected by double-deficiency of *Src-1* and *Src-2*. As noted, *Src-1/-2 dKO* mice succumbed at birth to alveolar collapse/atelectasis (98), and this suggested that surfactant glycerophospholipids may also be altered. In this regard, we found that amniotic fluid levels of the major and most surface-active surfactant component, DPPC, were significantly reduced in *Src-1/-2 dKO* fetuses compared to WT (70).

We also considered the role of the glycerophospholipid, PAF, which is proinflammatory, produced by the developing fetal lung together with surfactant lipids and SP-A and secreted into amniotic fluid near term. PAF, which activates leukocytes and stimulates their migration, was suggested to contribute to the initiation of term and preterm labor (68, 79, 99–101). PAF also directly stimulated the contraction of myometrial strips (102–106). Intriguingly, we observed that PAF levels in fetal lungs and amniotic fluid of *SRC-1/-2* double-deficient mice failed to increase toward term and were significantly reduced, compared to WT fetuses or those singly deficient in *Src-1* or *Src-2*.

**Figure 3:**
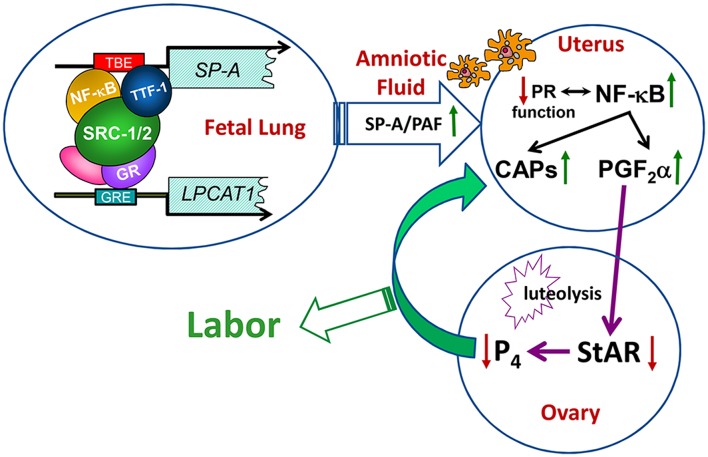
Transcriptional upregulation of the genes encoding SP-A and LPCAT1 in the fetal lung initiates a signaling cascade from fetus to mother that contributes to the initiation of parturition. Toward term, there is increased activation and binding of key transcription factors (e.g., thyroid transcription factor 1 and NF-κB) to response elements upstream of the *SP-*A gene, and of the glucocorticoid receptor (GR) to response elements upstream of the *LPCAT1* gene. These bound transcription factors recruit SRC-1 and SRC-2 to the promoters of these genes, resulting in increased *SP-A* and *LPCAT1* expression and the secretion of SP-A and PAF into amniotic fluid. These immune modulators activate immune cells that migrate into fetal membranes, decidua, and myometrium to increase production of proinflammatory cytokines leading to an activation of inflammatory transcription factors (e.g., NF-κB and AP-1). This contributes to the decline in PR function, with increased contractile (*CAP*) gene expression and synthesis of PGF_2_α, which circulates to the ovary to inhibit steroidogenic acute regulatory protein (StAR), promote luteolysis and cause a decline in circulating P_4_. The decrease in P_4_/PR function causes further upregulation of *CAP* gene expression and culminates in labor. Reproduced from Gao et al. (70).

Since both DPPC and PAF levels were significantly decreased in the amniotic fluid of *Src-1/-2* double-deficient fetuses, we searched for glycerophospholipid metabolizing enzymes that might coordinately regulate the synthesis of both of these molecules. In so doing, we discovered that lysophosphatidylcholine acyltransferase 1 (Lpcat1), which serves a key role in the deacylation/reacylation of the *sn-2* position of both DPPC and PAF to make the surface-active and pro-inflammatory molecules, respectively (107–109), was significantly decreased in lungs of *Src-1/-2* double-deficient fetuses compared to WT (70). Lpcat1 was previously found to be expressed specifically in mouse lung type II cells, developmentally-induced in fetal lung toward term, and stimulated by glucocorticosteroids (108). The role of Lpcat1 in surfactant synthesis by the developing lung was supported by the finding that mice carrying a hypomorphic allele of *Lpcat1* manifested atelectasis at birth and a deficiency in surfactant DPPC (107). Notably, the gestational increase of Lpcat1 was blocked in lungs of *Src-1/-2* double-deficient fetuses. Further, PAF or SP-A injection into the AF at 17.5 dpc rescued the parturition delay, enhanced uterine NF-κB activation and *CAP* gene expression and promoted luteolysis in *Src-1/2-*deficient mice (70). These collective findings further demonstrate the role of the fetal lung in producing signals for the initiation of labor when surfactant production is increased, and that SRC-1/2 coactivators serve crucial roles through enhanced production of SP-A and PAF (Figure 3).

## P_4_/PR Maintains Myometrial Quiescence via Concerted Mechanisms

**Figure 4:**
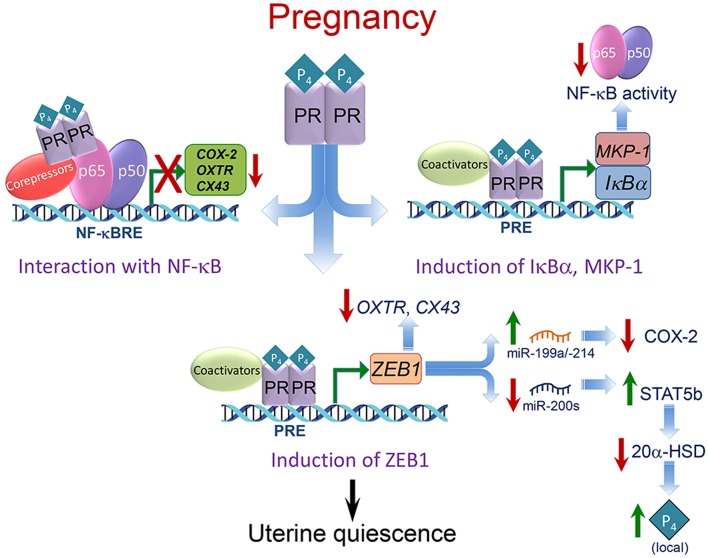
Mechanisms whereby P_4_/PR maintains myometrial quiescence during pregnancy. Multiple mechanisms mediate effects of P_4_/PR to suppress proinflammatory and *CAP* gene expression in the myometrium. As shown in the upper left, P_4_/PR exerts anti-inflammatory effects by tethering to NF-κB p65 bound to NF-κB response elements (NF-κBRE) within the promoters of proinflammatory and *CAP* genes. This stimulates the recruitment of corepressors, such as GATAD2B. Similar mechanisms are believed to occur at AP-1 sites upstream of *CAP* gene promoters, whereby PR interacts with FOS/JUN and recruits corepressors, such as P54^nrb^/Sin3A/HDAC. As shown in the upper right, P_4_/PR inhibits NF-κB activation and proinflammatory and *CAP* gene expression in myometrium by binding to the *I*κ*B*α promoter and enhancing IκBα expression. The increased cytoplasmic levels of IκBα interact with NF-κB to block its activation and translocation to the nucleus. P_4_/PR also exerts anti-inflammatory effects in myometrium by binding to the *MKP1*/*DUSP1* promoter to increase its expression and prevent MAPK activation, resulting in the inhibition of NF-κB and AP-1 activity. As shown in the lower panel, P_4_/PR blocks *CAP* gene expression by binding to the *ZEB1* promoter and enhancing its expression. The increased levels of ZEB1 bind to the promoters of *CAP* genes to inhibit their expression, activates expression of the *miR-199a/-214* cluster to inhibit expression of their target, COX-2, and inhibits miR-200 expression, leading to further induction of ZEB1 and ZEB2 as well as STAT5b, causing suppression of 20α-HSD and increased local P_4_ accumulation. Modified from Renthal et al. (58).

As described below, P_4_/PR maintains myometrial quiescence through a number of cooperative mechanisms. These include: (1) inhibiting transcriptional activity of the pro-inflammatory transcription factors, nuclear factor κB (NF-κB) (110) and activating protein 1 (AP-1) (111), via direct interaction and recruitment of corepressors (10, 111, 112); (2) inducing inhibitors of proinflammatory transcription factor activation (IκBα, MKP-1) (110, 113–115); (3) upregulating expression of transcriptional repressors of *CAP* genes (e.g., ZEB1) (56, 116) (Figure 4).

### P_4_/PR Has Anti-inflammatory Actions in the Myometrium

We (110, 114, 117) and others (46, 118–120) have obtained compelling evidence that P_4_/PR maintains myometrial quiescence through its action to block inflammation (Figure 4). Using hTERT-HM cells, we observed that P_4_/PR serves an anti-inflammatory role by antagonizing the activation of NF-κB and preventing the induction of COX-2 (117, 121), proinflammatory cytokines (10) and *CAP* genes (56). Using chromatin immunoprecipitation (ChIP), we found that P_4_ treatment of the hTERT-HM cells prevented interleukin-1 (IL-1)-induced binding of endogenous NF-κB p65 to NF-κB response elements in the *COX-2* and *IL-8* promoters (10, 110). The P_4_-mediated inhibition of proinflammatory and *CAP* gene transcription may be caused, in part, by the direct binding of PR to p65 (122) to inhibit NF-κB DNA-binding and transcriptional activity.

P_4_/PR can also block NF-κB activation and inflammation by increasing the expression of IκBα, which sequesters NF-κB in the cytoplasm and prevents its activation (110) (Figure 4). P_4_ treatment of hTERT-HM cells rapidly induced expression of IκBα, which preceded the effect of P_4_ to inhibit IL-1β-induced COX-2 expression (110). Moreover, P_4_ blocked the IL-1β-mediated decrease in IκBα protein, which suggested P_4_/PR inhibition of the proteasome pathway (123). Consequently, more NF-κB remains sequestered in the cytoplasm in an inactive state. Progesterone inhibition of NF-κB activation by upregulation of IκBα has also been reported in Mϕ (124) and breast cancer (113, 125) cell lines. The anti-inflammatory actions of P_4_/PR were further mediated by the upregulation of the mitogen-activated protein kinase (MAPK) inhibitor, MAPK phosphatase-1/dual specificity phosphatase 1 (MKP-1/DUSP1) (114, 115). Inhibition of MAPK, in turn, inhibits activation of NF-κB and AP-1 (126–128).

### PR Inhibits Proinflammatory and *CAP* Gene Expression by Recruitment of Corepressors

To define the mechanisms whereby P_4_ inhibits proinflammatory and *CAP* gene expression in the myometrium, we analyzed the capacity of PR_WT_ *vs*. various PR mutants to mediate P_4_ inhibition of proinflammatory gene expression in hTERT-HM human myometrial cells. These included a sumoylation mutant, a hinge domain mutation to prevent PR dimerization, and mutations of three amino acids in the DNA-binding domain (DBD). We observed that mutation of the PR-DBD had the most profound effect to prevent P_4_-inhibition of proinflammatory genes (10). Consequently, P_4_-mediated transrepression was significantly reduced in cells stably expressing a PR-A or PR-B DNA-binding domain mutant (PR_mDBD_), compared to cells expressing PR_WT_. ChIP analysis of the hTERT-HM cells revealed that P_4_/PR_WT_ transrepressive activity was associated with P_4_-induction of PR recruitment and inhibition of NF-κB p65 and RNA Pol II recruitment to an NF-κB response element in the *COX-2* and *IL-8* promoters. Importantly, in response to P_4_ treatment, equivalent recruitment of PR_WT_ and PR_mDBD_ to *COX-2* and *IL-8* promoters was observed. This suggested that the inhibitory effects of P_4_/PR on COX-2 and IL-8 expression were not mediated by direct DNA binding, but most likely by tethering to NF-κB. This led us to postulate that nuclear proteins interacting with the PR-DBD may mediate transrepression by P_4_/PR. Using immunoprecipitation, followed by mass spectrometry, we identified proteins that interacted strongly with PR_WT_ and weakly with the PR_DBD_ mutants. Among these was the transcriptional repressor, GATA Zinc Finger Domain Containing 2B (GATAD2B), which interacted with the PR-DBD and was required for P_4_/PR suppression of *proinflammatory* and *CAP* gene expression (10). Accordingly, P_4_ treatment of PR_WT_ hTERT-HM cells increased recruitment of endogenous GATAD2B to *COX-2* and *IL-8* promoters, whereas, siRNA knockdown of endogenous GATAD2B significantly reduced P_4_/PR_WT_ transrepression of *COX-2* and *IL-8*. Notably, GATAD2B expression decreased significantly in pregnant mouse and human myometrium during labor (10). Together, our findings suggest that GATAD2B serves as a novel mediator of P_4_/PR suppression of proinflammatory and *CAP* genes during pregnancy. Thus, the decline in GATAD2B expression near term may contribute to the loss of PR function leading to labor.

The induction of *CX43* expression by proinflammatory stimuli in the pregnant myometrium is mediated, in part, by increased transcriptional activity of members of the AP-1 family, which comprises Fos/Jun heterodimers or Jun/Jun homodimers. Fos/Jun heterodimers were found to be strong inducers of *CX43* expression compared to Jun/Jun homodimers, which were relatively weak (129). P_4_ acting through PR-B was found to repress *CX43* expression by recruiting inactive Jun/Jun homodimers and the P54^nrb^/Sin3A/HDAC corepressor complex to the *CX43* promoter (130). Near term, it was suggested that the increased metabolism of P_4_ by 20α-hydroxysteroid dehydrogenase (20α-HSD) in myometrium (131) and an inflammation-induced increase in the PR-A/PR-B ratio (8, 132, 133) caused PR-A to become free of ligands. The unliganded PR is then proposed to recruit relatively active Fra2/JunD heterodimers (129, 130), resulting in the activation of *CX43* expression. This switch may allow transformation of PR-A to an activator of *CX43* expression.

### P_4_/PR Maintains Myometrial Quiescence via Induction of ZEB1/2 and STAT5b

#### ZEB1 and ZEB2

Our findings reveal that P_4_/PR maintains myometrial quiescence, in part, by the induction of the zinc finger E-box-binding transcriptional repressor, ZEB1/TCF8/δEF1 (Figure 4), which binds to promoters of the *CAP* genes, *OXTR*, and *CX43* and the gene encoding members of the miR-200 family and represses their expression. Downregulation of miR-200s promotes further upregulation of ZEB1 and the related transcription factor, ZEB2/SIP2 (56) (Figure 2). Zeb1 was found to be expressed highly in mouse myometrium (116) and to be upregulated by P_4_/PR (134). We observed that the expression of Zeb1 and Zeb2 was elevated in myometrial tissues of 15.5 dpc pregnant mice and decreased precipitously toward term with the decline in circulating P_4_ and in PR function (56). Conversely, treatment of pregnant mice with RU486 or with the bacterial endotoxin, lipopolysaccharide (LPS), to induce preterm labor (56) caused significant inhibition of Zeb1/2 expression. ZEB1 and ZEB2 levels also were decreased in term myometrial tissues from women in labor, *vs*. tissues from women not in labor at term (56). ChIP-qPCR analysis of pregnant mouse myometrium revealed that endogenous Zeb1 bound to E-box-containing regions of the mouse *Cx43* and *Oxtr* promoters at relatively high levels at 15.5 dpc and declined markedly at term (56). Importantly, ZEB1 overexpression in human myometrial hTERT-HM cells caused a pronounced inhibition of OXTR and CX43 mRNA levels and blocked oxytocin-induced contraction of these cells using an *in vitro* contraction assay (56). Thus, the decrease in Zeb1 expression and binding to *CAP* gene promoters toward term likely promoted upregulation of *OXTR* and *CX43* leading to parturition.

To further assess the effects of P_4_ on Zeb expression, timed-pregnant mice were injected with P_4_ or vehicle daily between 15.5 and 18.5 dpc. P_4_ treatment, which delayed labor, caused a significant upregulation of Zeb1 and inhibited myometrial *CX43* and *OXTR* gene expression, compared to vehicle injected mice. A similar inductive effect of P_4_ on ZEB1 expression was observed in cultured T47D breast cancer cells. The stimulatory effect of P_4_ on ZEB1 expression was mediated at the level of gene transcription by the direct binding of PR to response element(s) within the ZEB1 promoter (56). Notably, ZEB2 is not directly regulated by P_4_/PR (56).

ZEB1 and ZEB2 are directly targeted by all five members of the highly conserved miR-200 family (135–138), which significantly increase in mouse and human myometrium toward term and in labor, in association with the decline in ZEB1 and ZEB2 expression (56). ZEB1 and ZEB2 also negatively regulate miR-200 expression. Accordingly, ZEB1/2 and miR-200s exist in a hormonally-regulated negative feedback loop (135–138). Thus, during pregnancy, elevated P_4_/PR increases the expression of ZEB1, which suppresses the miR-200 family, as well as contraction-associated genes. The decrease in miR-200 expression further upregulates ZEB1 and increases the expression of ZEB2. Near term, the decline in circulating P_4_ and/or PR function causes the downregulation of ZEB1 expression, and consequent upregulation of the miR-200 family, further suppressing ZEB1 and ZEB2. This de-represses *CAP* gene expression, resulting in increased uterine contractility and labor (Figure 2).

In parallel with our discovery of the gestational upregulation of the miR-200 family, we found that the conserved *miR-199a/-214 cluster* was significantly downregulated in mouse myometrium near term (56, 57), and in myometrial biopsies from women in-labor, compared to those not-in-labor at term (57). The *miR-199a/-214* cluster, comprised of miR-199a-5p, miR-199a-3p, and miR-214, are encoded within a 6-kb anti-sense transcript of the *Dynamin3* gene (*Dnm3os*), which is highly expressed in pregnant uterus (139). We observed that E_2_ treatment of ovariectomized mice suppressed, whereas P_4_ enhanced, uterine miR-199a-3p/-214 expression (57). Interestingly, these opposing hormonal effects were mediated by ZEB1, which, as described above, is induced by P_4_ (56, 116) and inhibited by E_2_ (57). ZEB1 binds directly to *the miR199a/-214* promoter to activate its transcription (57) (Figure 2). Importantly, miR-199a-3p and miR-214 both target the mRNA for COX-2 (57, 140). Thus, miR-199a-3p and miR-214 maintain uterine quiescence by suppressing the synthesis of contractile prostaglandins. These collective findings revealed the intriguing central role of ZEB1 as an inhibitor of the *miR-200* family and an inducer of the *miR-199a/-214 cluster*, which are oppositely regulated by P_4_ and E_2_ (Figure 2).

#### Signal Transducer and Activator of Transcription (STAT)5b

Increased local metabolism of P_4_ to inactive products near term in the uterus and cervix has been suggested to contribute to the decline in PR function that is crucial for the initiation of parturition in all mammals (19, 141–144). The finding that mice with a deletion of the gene encoding the P_4_-metabolizing enzyme, 20α-hydroxysteroid dehydrogenase (20α-HSD), manifested a significant delay in the timing of parturition, indicates its important role (20). In myometrium of pregnant women at term, a pronounced decrease in the ratio of P_4_ to 20α-dihydroprogesterone (20α-OHP), an inactive metabolite of P_4_ generated by 20α-HSD, were observed (144). In the course of our studies, we discovered that miR-200s directly target the P_4_-induced transcription factor STAT5b (131), which is a negative regulator of 20α-HSD in reproductive tissues (20, 145). Thus, the upregulation of miR-200 expression in mouse myometrium near term and in human myometrium during labor (56) was associated with suppression of STAT5b and induction of 20α-HSD (131). In contrast, throughout most of pregnancy, increased P_4_ levels upregulate ZEB1 and inhibit miR-200 expression in the myometrium (56). This, in turn, allows upregulation of STAT5b, which inhibits 20α-HSD expression (131) to maintain elevated endogenous levels of P_4_ and myometrial quiescence (Figure 2).

### P_4_/PR Induction of Caspases in the Pregnant Myometrium During Mid-Gestation Maintains Quiescence

During early to mid-pregnancy in rats there is a high rate of myometrial cell proliferation and hyperplasia, exemplified by increased BrdU incorporation and PCNA staining in the longitudinal smooth muscle layer (146). This declines precipitously by 17 dpc (term = 23 dpc) and is accompanied by an increase in cellular hypertrophy. From 12 to 15 dpc, there was a remarkable induction of the stress-induced caspase cascade (cleaved caspases 9, 3, 6, and 7) (146). However, this was not accompanied by evidence for apoptosis. Rather, it was suggested that caspase activation may cause inhibition of myometrial proliferative activity and promote the transition to hypertrophy and smooth muscle cell differentiation (146). Similar inductive changes in activation of the caspase cascade were observed in pregnant mouse myometrium from 12 to 15 dpc (term = 19 dpc) (147). Importantly, the activation of caspase 3 was found to be stimulated by P_4_ treatment. Moreover, caspase 3 activation was accompanied by the cleavage of myocyte contractile proteins, smooth muscle α- and γ-actins (147) as well as downregulation of the gap junction protein Cx43 (148). Thus, an important mechanism whereby P_4_/PR maintains myometrial quiescence during pregnancy is via its action to induce the active caspase cascade and cause degradation of proteins involved in myometrial contractility. The decline in PR function in the pregnant myometrium toward term results in decreased caspase activation and allows for the increase in contractile protein accumulation. Notably, caspase activation in uterine myocytes was also associated with induction of the endoplasmic reticulum stress response (ERSR), which is likely enhanced by physiological/mechanical stimuli (149). The ERSR was reduced near term by upregulation of the adaptive unfolded protein response (UPR), resulting in a decline in active caspase 3 and for the induction of contractile proteins (149).

## Mechanisms for the Decline in PR Function Leading to Parturition

The decline in PR function leading to parturition, which is fundamental and critical for species survival, is mediated by multiple complementary mechanisms, several of which are discussed below. It is likely that all of these processes are regulated by an increased inflammatory response within the myometrium at term, resulting in activation of NF-κB and AP-1 transcription factors. In various cell types, activation of NF-κB represses PR transcriptional activity, while PR activation also represses NF-κB-mediated transcription (28, 122).

### Altered PR Isoform Expression and Posttranslational Modification in the Myometrium Toward Term

As mentioned, PR-A, which is truncated at its N-terminus, contains only two of the three transcriptional activation domains that are present in PR-B. Thus, in certain cell- and gene-specific contexts, including human myometrial cells, PR-A has been found to repress PR-B transcriptional activity (5–7). PR-A and PR-B are differentially regulated in the human myometrium during pregnancy (8); the ratio of PR-A to PR-B mRNA (9) and protein (7) was observed to be significantly higher in term myometrium from women in labor compared to those not in labor. Moreover, in hTERT-HM myometrial cells stably expressing PR-A or PR-B, it was observed that P_4_ had decreased anti-inflammatory activity in PR-A-expressing cells when compared to those expressing PR-B (10). Furthermore, proinflammatory stimuli specifically increased phosphorylation of the PR-A isoform on Ser-344/345 in a P_4_-dependent manner and enhanced its ability to antagonize the anti-inflammatory activity of PR-B (133). Notably, in term myometrium from women in-labor vs. not-in-labor, phosphorylation of Ser-345 occurred exclusively on PR-A, and the abundance of phospho-Ser-345-PR-A relative to total PR-A was increased significantly in laboring vs. non-laboring myometrium in association with increased NF-κB activation (133).

A third PR isoform, PR-C (~60 kDa), which is truncated from the N-terminus and lacks part of the DNA binding domain, is primarily cytoplasmic in its localization (150, 151). Since PR-C can bind P_4_ (152), it may inhibit PR function by sequestering P_4_ and/or by physically interacting with PR-B to reduce its DNA-binding capacity (150). In fundal myometrium from women before and after the initiation of labor at term, we observed a labor-associated increase in PR-C mRNA and a ~60 kDa immunoreactive PR protein. This was temporally and spatially associated with activation of NF-κB (27). A temporal increase in a truncated 60 kDa PR isoform was also observed in mouse uterus near term. Notably, the identity of truncated PR isoforms in the pregnant uterus (8, 153) requires further study.

### Decreased Expression of Selected Coregulators in the Myometrium Toward Term

P_4_/PR activation of target gene expression is dependent upon recruitment of coactivator complexes, which contain histone acetyltransferases and cause an opening of chromatin structure (154, 155). Previously, we observed that expression of selected steroid receptor coactivators (SRC) and the histone acetyltransferase, CREB-binding protein (CBP), declined in myometrium of women in labor, compared to non-laboring myometrium, and were profoundly decreased in myometrial tissues of pregnant mice at term (156). This was associated with decreased levels of acetylated histone H3. Remarkably, treatment of pregnant mice with the histone deacetylase (HDAC) inhibitor, trichostatin A (TSA), during late gestation increased myometrial histone acetylation and delayed parturition by 24–48 h (156). Subsequently, it was reported that HDAC inhibitors suppressed proinflammatory gene expression in cultured human myometrial cells (157) and strongly inhibited contractility of human myometrial strips (158). Collectively, these findings suggest that decreased expression of PR coactivators and in histone acetylation in the myometrium during late gestation may impair the capacity of P_4_/PR to upregulate genes that maintain myometrial quiescence and increase sensitivity of the uterus to prostaglandins and other contractile factors. In cultured human myometrial cells, the effect of TNFα to inhibit SRC-1 and SRC-2 expression mediated TNFα inhibition of PR-B transcriptional activity (159). Thus, the decline in coactivators in the myometrium near term may be caused by induction of proinflammatory mediators. Moreover, corepressors, GATAD2B (10) and p54nrb (non-POU-domain-containing, octamer binding protein) (111), which interact with PR and mediate its capacity to repress proinflammatory and *CAP* gene expression, were found to decrease in rodent myometrium at term, further contributing to the decline in PR function.

### Increased Metabolism of P_4_ Toward Term Contributes to the Decline in PR Function Leading to Parturition

As mentioned, in women, circulating P_4_ levels remain elevated throughout pregnancy and labor due to the maintenance of placental P_4_ production (160). Furthermore, levels of PR remain elevated in reproductive tissues during pregnancy and into labor (12). Even in rodents, where P_4_ production by the corpus luteum declines precipitously near term, the levels of P_4_ in circulation remain higher than the K_d_ for binding to PR (17). As mentioned, in the myometrium of pregnant women at term, there is a pronounced decrease in the ratio of P_4_ to 20α-dihydroprogesterone (144), an inactive P_4_ metabolite generated by the enzyme 20α-HSD. In mice, the initiation of parturition is accompanied by increased expression of the P_4_-metabolizing enzymes, 20α-HSD in the uterus (131) and 5α-reductase type I in the cervix (18, 19). Accordingly, gene-targeted mice lacking 5α-reductase type I fail to deliver because of impaired cervical ripening, even though maternal circulating P_4_ levels decline normally (18, 19). Similarly, *20*α*-HSD* knockout mice manifested severely delayed parturition (20, 161). Thus, increased local metabolism of P_4_ in the uterus and cervix near term contributes to the decline in PR function and is crucial for the initiation of parturition (19, 141–144).

## Conclusions

Throughout pregnancy, the critical role of P_4_/PR in maintaining myometrial quiescence is principally mediated by its capacity to inhibit inflammatory pathways and to suppress *CAP* gene expression. As depicted in Figure 4, this occurs via several cooperative mechanisms that include: tethering of PR to the inflammatory transcription factors, NF-κB or AP-1, with recruitment of corepressors (10, 110–112, 130); PR promoter binding and transcriptional activation of genes encoding the NF-κB suppressor, IκBα (110), and the MAPK inhibitor, MKP1 (114). Increased P_4_/PR also upregulates expression of transcription factor ZEB1, which interacts directly with the promoters of the *OXTR* and *CX43* genes to inhibit their expression and suppresses expression of the miR-200 family (56). Decreased miR-200 expression allows upregulation of its target, STAT5b, a transcriptional inhibitor of *20*α*-HSD*, so that P_4_ metabolism in the myometrium is prevented (131). The increased ZEB1 also upregulates the expression of members of the miR-199a/-214 cluster, which directly target COX-2, resulting in the suppression of contractile prostaglandin synthesis (57) (Figures 2, 4). P_4_ also contributes to myometrial quiescence by activation of the caspase cascade, which maintains low levels of myocyte contractile proteins through increased caspase-mediated degradation (147).

The transition of the pregnant myometrium to an inflammatory, contractile state at term is affected by cooperative signals from the mother and fetus (Figure 1). Our findings suggest that appropriate timing for parturition is mediated, in part, by the induction of surfactant signals, SP-A and PAF, produced by the fetal lung and secreted into amniotic fluid where they interact with fetal Mϕ to alter their phenotypic state (23, 69, 70). These activated immune cells then migrate to the maternal uterus (23) where they promote an inflammatory response with activation of NF-κB and AP-1, leading to a decline in PR function and increased expression of proinflammatory and *CAP* genes in the myometrium (Figures 1, 3). The decline in PR function in human myometrium is thought to be mediated by: an inflammation-induced increase in PR-A, relative to PR-B isoform expression (133), with possible upregulation of other truncated PR isoforms (27); a decreased expression of ZEB1 and coordinate induction of miR-200 family expression (56), resulting in suppression of STAT5b, upregulation of 20α-HSD and with increased local metabolism of P_4_ (131); the direct interaction of NF-κB p65 with PR (122); a decline in PR coactivators (156). The decline in ZEB1 also contributes to upregulation of *OXTR* and *CX43* (56) and a decrease in *miR-199a/-214* expression with the induction of their target, COX-2 (57), and of PGF_2_α synthesis (Figure 1). Toward term, the increase in E_2_ production and ERα activation in the myometrium results in increased proinflammatory and *CAP* gene expression, which is mediated, in part, by inhibition of ZEB1/2 (57) and by the decreased expression of miR-181b, allowing for the upregulation of its targets, ERα, TNFα and c-FOS (53). These highly coordinated molecular events, together with increased myometrial stretch and a decline in PR corepressor expression culminate in the increased myometrial contractility leading to parturition.

Our extensive knowledge of the mechanisms that underlie myometrial quiescence during pregnancy and its transition to a contractile state prior to parturition has led to the identification of a number of conserved potential therapeutic targets for the prevention of preterm labor and its consequences. Several of these targets include miRNA clusters and families that are coordinately upregulated or downregulated toward term and target a number of signaling molecules and pathways. Of considerable importance is the miR-200 family, which is markedly upregulated in pregnant human and mouse myometrium toward term. miR-200 family members target and downregulate expression of ZEB1 and ZEB2, leading to increased contractile gene expression and the suppression of STAT5b, which results in increased local metabolism of P_4_ by 20α-HSD (131). The decline in ZEB1/2 also causes decreased miR-199a and miR-214 expression, which both independently target the contractile gene, *PTGS2/COX2*, resulting in an increase in prostaglandin synthesis (57). The induction of miR-200s also directly targets PR (162), which may contribute to the loss of its function. Thus, anti-miR-200 therapy could form the basis for a comprehensive, multifactorial and highly effective therapeutic strategy for prevention of preterm birth.

## Author Contributions

CM wrote the manuscript. LG and AM critiqued and edited the manuscript.

### Conflict of Interest

The authors declare that the research was conducted in the absence of any commercial or financial relationships that could be construed as a potential conflict of interest.

## References

[1] Blencowe H Cousens S Chou D Oestergaard M Say L Moller AB. Born too soon: the global epidemiology of 15 million preterm births. Reprod Health. (2013) 10(Suppl 1):S2. 10.1186/1742-4755-10-S1-S2 24625129 PMC3828585

[2] Peltier MR Drobek CO Bhat G Saade G Fortunato SJ Menon R. Amniotic fluid and maternal race influence responsiveness of fetal membranes to bacteria. J Reprod Immunol. (2012) 96:68–78. 10.1016/j.jri.2012.07.006 23021257 PMC3596009

[3] Kastner P Bocquel MT Turcotte B Garnier JM Horwitz KB Chambon P Transient expression of human and chicken progesterone receptors does not support alternative translational initiation from a single mRNA as the mechanism generating two receptor isoforms. J Biol Chem. (1990) 265:12163–7. 2373686

[4] Conneely OM Maxwell BL Toft DO Schrader WT O'Malley BW. The A and B forms of the chicken progesterone receptor arise by alternate initiation of translation of a unique mRNA. Biochem Biophys Res Commun. (1987) 149:493–501. 10.1016/0006-291X(87)90395-0 3426587

[5] Giangrande PH Kimbrel EA Edwards DP McDonnell DP. The opposing transcriptional activities of the two isoforms of the human progesterone receptor are due to differential cofactor binding. Mol Cell Biol. (2000) 20:3102–15. 10.1128/MCB.20.9.3102-3115.2000 10757795 PMC85605

[6] Vegeto E Shahbaz MM Wen DX Goldman ME O'Malley BW McDonnell DP. Human progesterone receptor A form is a cell- and promoter-specific repressor of human progesterone receptor B function. Mol Endocrinol. (1993) 7:1244–55. 10.1210/mend.7.10.8264658 8264658

[7] Pieber D Allport VC Hills F Johnson M Bennett PR. Interactions between progesterone receptor isoforms in myometrial cells in human labour. Mol Hum Reprod. (2001) 7:875–9. 10.1093/molehr/7.9.875 11517295

[8] Merlino AA Welsh TN Tan H Yi LJ Cannon V Mercer BM. Nuclear progesterone receptors in the human pregnancy myometrium: evidence that parturition involves functional progesterone withdrawal mediated by increased expression of progesterone receptor-A. J Clin Endocrinol Metab. (2007) 92:1927–33. 10.1210/jc.2007-0077 17341556

[9] Mesiano S Chan EC Fitter JT Kwek K Yeo G Smith R. Progesterone withdrawal and estrogen activation in human parturition are coordinated by progesterone receptor A expression in the myometrium. J Clin Endocrinol Metab. (2002) 87:2924–30. 10.1210/jcem.87.6.8609 12050275

[10] Chen CC Montalbano AP Hussain I Lee WR Mendelson CR. The transcriptional repressor GATAD2B mediates progesterone receptor suppression of myometrial contractile gene expression. J Biol Chem. (2017) 292:12560–76. 10.1074/jbc.M117.791350 28576827 PMC5535031

[11] Virgo BB Bellward GD. Serum progesterone levels in the pregnant and postpartum laboratory mouse. Endocrinology. (1974) 95:1486–90. 10.1210/endo-95-5-1486 4473330

[12] Challis JRG Matthews SG Gibb W Lye SJ. Endocrine and paracrine regulation of birth at term and preterm. Endocr Rev. (2000) 21:514–50. 10.1210/er.21.5.514 11041447

[13] Frydman R Lelaidier C Baton-Saint-Mleux C Fernandez H Vial M Bourget P. Labor induction in women at term with mifepristone (RU 486): a double-blind, randomized, placebo-controlled study. Obstet Gynecol. (1992) 80:972–5. 10.1016/0020-7292(93)90660-O 1448266

[14] Elliott CL Brennand JE Calder AA. The effects of mifepristone on cervical ripening and labor induction in primigravidae. Obstet Gynecol. (1998) 92:804–9. 10.1097/00006250-199811000-00013 9794673

[15] Stenlund PM Ekman G Aedo AR Bygdeman M. Induction of labor with mifepristone–a randomized, double-blind study versus placebo. Acta Obstet Gynecol Scand. (1999) 78:793–8. 10.1080/j.1600-0412.1999.780910.x 10535343

[16] Chwalisz K. The use of progesterone antagonists for cervical ripening and as an adjunct to labour and delivery. Hum Reprod. (1994) 9(Suppl 1):131–61. 10.1093/humrep/9.suppl_1.131 7962460

[17] Pointis G Rao B Latreille MT Mignot TM Cedard L. Progesterone levels in the circulating blood of the ovarian and uterine veins during gestation in the mouse. Biol Reprod. (1981) 24:801–5. 10.1095/biolreprod24.4.801 7248413

[18] Mahendroo MS Cala KM Russell DW 5α-reduced androgens play a key role in murine parturition. Mol Endocrinol. (1996) 10:380–92. 10.1210/mend.10.4.8721983 8721983

[19] Mahendroo MS Porter A Russell DW Word RA. The parturition defect in steroid 5α-reductase type 1 knockout mice is due to impaired cervical ripening. Mol Endocrinol. (1999) 13:981–92. 10.1210/mend.13.6.0307 10379896

[20] Piekorz RP Gingras S Hoffmeyer A Ihle JN Weinstein Y. Regulation of progesterone levels during pregnancy and parturition by signal transducer and activator of transcription 5 and 20alpha-hydroxysteroid dehydrogenase. Mol Endocrinol. (2005) 19:431–40. 10.1210/me.2004-0302 15471942

[21] Cox SM Casey ML MacDonald PC. Accumulation of interleukin-1beta and interleukin-6 in amniotic fluid: a sequela of labour at term and preterm. Hum Reprod Update. (1997) 3:517–27. 10.1093/humupd/3.5.517 9528914

[22] Thomson AJ Telfer JF Young A Campbell S Stewart CJ Cameron IT. Leukocytes infiltrate the myometrium during human parturition: further evidence that labour is an inflammatory process. Hum Reprod. (1999) 14:229–36. 10.1093/humrep/15.1.229 10374126

[23] Condon JC Jeyasuria P Faust JM Mendelson CR. Surfactant protein secreted by the maturing mouse fetal lung acts as a hormone that signals the initiation of parturition. Proc Natl Acad Sci USA. (2004) 101:4978–83. 10.1073/pnas.0401124101 15044702 PMC387359

[24] Osman I Young A Ledingham MA Thomson AJ Jordan F Greer IA. Leukocyte density and pro-inflammatory cytokine expression in human fetal membranes, decidua, cervix and myometrium before and during labour at term. Mol Hum Reprod. (2003) 9:41–5. 10.1093/molehr/gag001 12529419

[25] Mendelson CR Montalbano AP Gao L. Fetal-to-maternal signaling in the timing of birth. J Steroid Biochem Mol Biol. (2017) 170:19–27. 10.1016/j.jsbmb.2016.09.006 27629593 PMC5346347

[26] Romero R Espinoza J Goncalves LF Kusanovic JP Friel L Hassan S. The role of inflammation and infection in preterm birth. Semin Reprod Med. (2007) 25:21–39. 10.1055/s-2006-956773 17205421 PMC8324073

[27] Condon JC Hardy DB Kovaric K Mendelson CR Upregulation of the progesterone receptor (PR)-C isoform in laboring myometrium by activation of NF-kappaB may contribute to the onset of labor through inhibition of PR function. Mol Endocrinol. (2006) 20:764–75. 10.1210/me.2005-0242 16339279

[28] Allport VC Pieber D Slater DM Newton R White JO Bennett PR. Human labour is associated with nuclear factor-kappaB activity which mediates cyclo-oxygenase-2 expression and is involved with the 'functional progesterone withdrawal'. Mol Hum Reprod. (2001) 7:581–6. 10.1093/molehr/7.6.581 11385114

[29] Lee YS Terzidou V Lindstrom T Johnson M Bennett PR. The role of CCAAT/enhancer-binding protein beta in the transcriptional regulation of COX-2 in human amnion. Mol Hum Reprod. (2005) 11:853–8. 10.1093/molehr/gah194 16399783

[30] Elliott CL Allport VC Loudon JA Wu GD Bennett PR Nuclear factor-κB is essential for up-regulation of interleukin-8 expression in human amnion and cervical epithelial cells. Mol Hum Reprod. (2001) 7:787–90. 10.1093/molehr/7.8.787 11470867

[31] Olson DM. The role of prostaglandins in the initiation of parturition. Best Pract Res Clin Obstet Gynaecol. (2003) 17:717–30. 10.1016/S1521-6934(03)00069-5 12972010

[32] Chow L Lye SJ. Expression of the gap junction protein connexin-43 is increased in the human myometrium toward term and with the onset of labor. Am J Obstet Gynecol. (1994) 170:788–95. 10.1016/S0002-9378(94)70284-5 8141203

[33] Fuchs AR Fuchs F Husslein P Soloff MS. Oxytocin receptors in the human uterus during pregnancy and parturition. Am J Obstet Gynecol. (1984) 150:734–41. 10.1016/0002-9378(84)90677-X 6093538

[34] Soloff MS Cook DL Jr Jeng YJ Anderson GD. *In situ* analysis of interleukin-1-induced transcription of *COX-2* and *IL-8* in cultured human myometrial cells. Endocrinology. (2004) 145:1248–54. 10.1210/en.2003-1310 14645117

[35] Rauk PN Chiao JP. Interleukin-1 stimulates human uterine prostaglandin production through induction of cyclooxygenase-2 expression. Am J Reprod Immunol. (2000) 43:152–9. 10.1111/j.8755-8920.2000.430304.x 10735591

[36] Shynlova O Tsui P Dorogin A Lye SJ. Monocyte chemoattractant protein-1 (CCL-2) integrates mechanical and endocrine signals that mediate term and preterm labor. J Immunol. (2008) 181:1470–9. 10.4049/jimmunol.181.2.1470 18606702

[37] Sooranna SR Lee Y Kim LU Mohan AR Bennett PR Johnson MR. Mechanical stretch activates type 2 cyclooxygenase via activator protein-1 transcription factor in human myometrial cells. Mol Hum Reprod. (2004) 10:109–13. 10.1093/molehr/gah021 14742695

[38] Goldenberg RL Iams JD Miodovnik M Van Dorsten JP Thurnau G Bottoms S. The preterm prediction study: risk factors in twin gestations. National Institute of Child Health and Human Development Maternal-Fetal Medicine Units Network. Am J Obstet Gynecol. (1996) 175:1047–53. 10.1016/S0002-9378(96)80051-2 8885774

[39] Esplin MS Peltier MR Hamblin S Smith S Fausett MB Dildy GA. Monocyte chemotactic protein-1 expression is increased in human gestational tissues during term and preterm labor. Placenta. (2005) 26:661–71. 10.1016/j.placenta.2004.09.012 16085045

[40] Adams Waldorf KM Singh N Mohan AR Young RC Ngo L Das A. Uterine overdistention induces preterm labor mediated by inflammation: observations in pregnant women and nonhuman primates. Am J Obstet Gynecol. (2015) 213:830. 10.1016/j.ajog.2015.08.028 26284599 PMC4679421

[41] Hua R Pease JE Sooranna SR Viney JM Nelson SM Myatt L. Stretch and inflammatory cytokines drive myometrial chemokine expression via NF-kappaB activation. Endocrinology. (2012) 153:481–91. 10.1210/en.2011-1506 22045664

[42] Kelly RW Carr GG Riley SC. The inhibition of synthesis of a beta-chemokine, monocyte chemotactic protein-1 (MCP-1) by progesterone. Biochem Biophys Res Commun. (1997) 239:557–61. 10.1006/bbrc.1997.7502 9344869

[43] Challis JR. Sharp increase in free circulating oestrogens immediately before parturition in sheep. Nature. (1971) 229:208. 10.1038/229208a0 4923273

[44] Buster JE Chang RJ Preston DL Elashoff RM Cousins LM Abraham GE Interrelationships of circulating maternal steroid concentrations in third trimester pregnancies. II. C_18_ and C_19_ steroids: estradiol, estriol, dehydroepiandrosterone, dehydroepiandrosterone sulfate, Δ^5^-androstenediol, Δ^4^-androstenedione, testosterone, and dihydrotestosterone. J Clin Endocrinol Metab. (1979) 48:139–42. 10.1210/jcem-48-1-139 154525

[45] Wu WX Myers DA Nathanielsz PW. Changes in estrogen receptor messenger ribonucleic acid in sheep fetal and maternal tissues during late gestation and labor. Am J Obstet Gynecol. (1995) 172:844–50. 10.1016/0002-9378(95)90009-8 7892873

[46] Tibbetts TA Conneely OM O'Malley BW. Progesterone via its receptor antagonizes the pro-inflammatory activity of estrogen in the mouse uterus. Biol Reprod. (1999) 60:1158–65. 10.1095/biolreprod60.5.1158 10208978

[47] Murata T Narita K Honda K Matsukawa S Higuchi T. Differential regulation of estrogen receptor alpha and beta mRNAs in the rat uterus during pregnancy and labor: possible involvement of estrogen receptors in oxytocin receptor regulation. Endocr J. (2003) 50:579–87. 10.1507/endocrj.50.579 14614214

[48] Piersanti M Lye SJ. Increase in messenger ribonucleic acid encoding the myometrial gap junction protein, connexin-43, requires protein synthesis and is associated with increased expression of the activator protein-1, c-fos. Endocrinology. (1995) 136:3571–8. 10.1210/endo.136.8.7628395 7628395

[49] Tsuboi K Sugimoto Y Iwane A Yamamoto K Yamamoto S Ichikawa A. Uterine expression of prostaglandin H2 synthase in late pregnancy and during parturition in prostaglandin F receptor-deficient mice. Endocrinology. (2000) 141:315–24. 10.1210/en.141.1.315 10614653

[50] Engstrom T. The regulation by ovarian steroids of prostaglandin synthesis and prostaglandin-induced contractility in non-pregnant rat myometrium. Modulating effects of isoproterenol. J Endocrinol. (2001) 169:33–41. 10.1677/joe.0.1690033 11250644

[51] Gibb W. The role of prostaglandins in human parturition. Ann Med. (1998) 30:235–41. 10.3109/07853899809005850 9677008

[52] Kushner PJ Agard DA Greene GL Scanlan TS Shiau AK Uht RM. Estrogen receptor pathways to AP-1. J Steroid Biochem Mol Biol. (2000) 74:311–7. 10.1016/S0960-0760(00)00108-4 11162939

[53] Gao L Wang G Liu W Kinser H Franco HL Mendelson CR. Reciprocal feedback between miR-181a and E_2_/ERα in myometrium enhances inflammation leading to labor. J Clin Endocrinol Metab. (2016) 101:3646–56. 10.1210/jc.2016-2078 27459534 PMC5052345

[54] Mitchell JA Lye SJ. Differential expression of activator protein-1 transcription factors in pregnant rat myometrium. Biol Reprod. (2002) 67:240–6. 10.1095/biolreprod67.1.240 12080023

[55] Wu C Gong Y Yuan J Zhang W Zhao G Li H. microRNA-181a represses ox-LDL-stimulated inflammatory response in dendritic cell by targeting c-Fos. J Lipid Res. (2012) 53:2355–63. 10.1194/jlr.M028878 22956783 PMC3466004

[56] Renthal NE Chen CC Williams KC Gerard RD Prange-Kiel J Mendelson CR. miR-200 family and targets, ZEB1 and ZEB2, modulate uterine quiescence and contractility during pregnancy and labor. Proc Natl Acad Sci USA. (2010) 107:20828–33. 10.1073/pnas.1008301107 21079000 PMC2996411

[57] Williams KC Renthal NE Gerard RD Mendelson CR. The microRNA (miR)-199a/214 cluster mediates opposing effects of progesterone and estrogen on uterine contractility during pregnancy and labor. Mol Endocrinol. (2012) 26:1857–67. 10.1210/me.2012-1199 22973051 PMC3487626

[58] Renthal NE Williams KC Montalbano AP Chen CC Gao L Mendelson CR. Molecular regulation of parturition: a myometrial perspective. Cold Spring Harb Perspect Med. (2015) 5:181–96. 10.1101/cshperspect.a023069 26337112 PMC4632865

[59] Liggins GC Fairclough RJ Grieves SA Kendall JZ Knox BS. The mechanism of initiation of parturition in the ewe. Recent Prog Horm Res. (1973) 29:111–59. 10.1016/B978-0-12-571129-6.50007-5 4356273

[60] Liggins GC. Premature delivery of foetal lambs infused with glucocorticoids. J Endocrinol. (1969) 45:515–23. 10.1677/joe.0.0450515 5366112

[61] Rainey WE Rehman KS Carr BR. The human fetal adrenal: making adrenal androgens for placental estrogens. Semin Reprod Med. (2004) 22:327–36. 10.1055/s-2004-861549 15635500

[62] Robinson BG Arbiser JL Emanuel RL Majzoub JA. Species-specific placental corticotropin releasing hormone messenger RNA and peptide expression. Mol Cell Endocrinol. (1989) 62:337–41. 10.1016/0303-7207(89)90022-1 2787253

[63] Florio P Cobellis L Woodman J Severi FM Linton EA Petraglia F. Levels of maternal plasma corticotropin-releasing factor and urocortin during labor. J Soc Gynecol Investig. (2002) 9:233–7. 10.1177/107155760200900409 12113883

[64] Torricelli M Giovannelli A Leucci E De Falco G Reis FM Imperatore A. Labor (term and preterm) is associated with changes in the placental mRNA expression of corticotrophin-releasing factor. Reprod Sci. (2007) 14:241–5. 10.1177/1933719107300971 17636237

[65] Keegan CE Herman JP Karolyi IJ O'Shea KS Camper SA Seasholtz AF. Differential expression of corticotropin-releasing hormone in developing mouse embryos and adult brain. Endocrinology. (1994) 134:2547–55. 10.1210/en.134.6.2547 8194481

[66] Muglia LJ Bae DS Brown TT Vogt SK Alvarez JG Sunday ME. Proliferation and differentiation defects during lung development in corticotropin-releasing hormone-deficient mice. Am J Respir Cell Mol Biol. (1999) 20:181–8. 10.1165/ajrcmb.20.2.3381 9922208

[67] Lopez BA Newman GE Phizackerley PJ Turnbull AC Surfactant stimulates prostaglandin E production in human amnion. Br J Obstet Gynaecol. (1988) 95:1013–7. 10.1111/j.1471-0528.1988.tb06506.x 3191038

[68] Frenkel RA Muguruma K Johnston JM. The biochemical role of platelet-activating factor in reproduction. Prog Lipid Res. (1996) 35:155–68. 10.1016/0163-7827(96)00002-1 8944225

[69] Montalbano AP Hawgood S Mendelson CR. Mice deficient in surfactant protein A (SP-A) and SP-D or in TLR2 manifest delayed parturition and decreased expression of inflammatory and contractile genes. Endocrinology. (2013) 154:483–98. 10.1210/en.2012-1797 23183169 PMC3529364

[70] Gao L Rabbitt EH Condon JC Renthal NE Johnston JM Mitsche MA. Steroid receptor coactivators 1 and 2 mediate fetal-to-maternal signaling that initiates parturition. J Clin Invest. (2015) 125:2808–24. 10.1172/JCI78544 26098214 PMC4563678

[71] Whitsett JA Weaver TE. Hydrophobic surfactant proteins in lung function and disease. N Engl J Med. (2002) 347:2141–8. 10.1056/NEJMra022387 12501227

[72] Kuroki Y Takahashi M Nishitani C. Pulmonary collectins in innate immunity of the lung. Cell Microbiol. (2007) 9:1871–9. 10.1111/j.1462-5822.2007.00953.x 17490408

[73] Hawgood S Shiffer K. Structures and properties of the surfactant-associated proteins. Annu Rev Physiol. (1991) 53:375–94. 10.1146/annurev.ph.53.030191.002111 2042965

[74] Wright JR. Immunoregulatory functions of surfactant proteins. Nat Rev Immunol. (2005) 5:58–68. 10.1038/nri1528 15630429

[75] Crouch E Wright JR. Surfactant proteins A and D and pulmonary host defense. Annu Rev Physiol. (2001) 63:521–54. 10.1146/annurev.physiol.63.1.521 11181966

[76] Kremlev SG Phelps DS. Surfactant protein A stimulation of inflammatory cytokine and immunoglobulin production. Am J Physiol Lung Cell Mol Physiol. (1994) 267:L712–9. 10.1152/ajplung.1994.267.6.L712 7810675

[77] Phelps DS. Surfactant regulation of host defense function in the lung: a question of balance. Pediatr Pathol Mol Med. (2001) 20:269–92. 10.3109/15513810109168822 11486734

[78] Mitchell MD MacDonald PC Casey ML. Stimulation of prostaglandin E2 synthesis in human amnion cells maintained in monolayer culture by a substance(s) in amniotic fluid. Prostaglandins Leukot Med. (1984) 15:399–407. 10.1016/0262-1746(84)90138-0 6593749

[79] Toyoshima K Narahara H Furukawa M Frenkel RA Johnston JM. Platelet-activating factor. Role in fetal lung development and relationship to normal and premature labor. Clin Perinatol. (1995) 22:263–80. 10.1016/S0095-5108(18)30285-9 7671539

[80] Mendelson CR Boggaram V. Hormonal control of the surfactant system in fetal lung. Annu Rev Physiol. (1991) 53:415–40. 10.1146/annurev.ph.53.030191.002215 2042967

[81] Korfhagen TR Bruno MD Glasser SW Ciraolo PJ Whitsett JA Lattier DL. Murine pulmonary surfactant SP-A gene: cloning, sequence, and transcriptional activity. Am J Physiol Lung Cell Mol Physiol. (1992) 263:L546–54. 10.1152/ajplung.1992.263.5.L546 1443158

[82] Alcorn JL Hammer RE Graves KR Smith ME Maika SD Michael LF. Analysis of genomic regions involved in regulation of the rabbit *surfactant protein A* gene in transgenic mice. Am J Physiol Lung Cell Mol Physiol. (1999) 277:L349–61. 10.1152/ajplung.1999.277.2.L349 10444530

[83] Kim CJ Kim JS Kim YM Cushenberry E Richani K Espinoza J Fetal macrophages are not present in the myometrium of women with labor at term. Am J Obstet Gynecol. (2006) 195:829–33. 10.1016/j.ajog.2006.06.052 16949420

[84] Lee DC Romero R Kim CJ Chaiworapongsa T Tarca AL Lee J. Surfactant protein-A as an anti-inflammatory component in the amnion: implications for human pregnancy. J Immunol. (2010) 184:6479–91. 10.4049/jimmunol.0903867 20439915 PMC3103775

[85] Gardai SJ Xiao YQ Dickinson M Nick JA Voelker DR Greene KE By binding SIRPalpha or calreticulin/CD91, lung collectins act as dual function surveillance molecules to suppress or enhance inflammation. Cell. (2003) 115:13–23. 10.1016/S0092-8674(03)00758-X 14531999

[86] Murakami S Iwaki D Mitsuzawa H Sano H Takahashi H Voelker DR. Surfactant protein A inhibits peptidoglycan-induced tumor necrosis factor-α secretion in U937 cells and alveolar macrophages by direct interaction with toll-like receptor 2. J Biol Chem. (2002) 277:6830–7. 10.1074/jbc.M106671200 11724772

[87] Sato M Sano H Iwaki D Kudo K Konishi M Takahashi H Direct binding of Toll-like receptor 2 to zymosan, and zymosan-induced NF-kappaB activation and TNF-alpha secretion are down-regulated by lung collectin surfactant protein A. J Immunol. (2003) 171:417–25. 10.4049/jimmunol.171.1.417 12817025

[88] Ohya M Nishitani C Sano H Yamada C Mitsuzawa H Shimizu T. Human pulmonary surfactant protein D binds the extracellular domains of Toll-like receptors 2 and 4 through the carbohydrate recognition domain by a mechanism different from its binding to phosphatidylinositol and lipopolysaccharide. Biochemistry. (2006) 45:8657–64. 10.1021/bi060176z 16834340

[89] Tsan MF Gao B. Endogenous ligands of Toll-like receptors. J Leukoc Biol. (2004) 76:514–9. 10.1189/jlb.0304127 15178705

[90] Shynlova O Tsui P Jaffer S Lye SJ. Integration of endocrine and mechanical signals in the regulation of myometrial functions during pregnancy and labour. Eur J Obstet Gynecol Reprod Biol. (2009) 144(Suppl 1):S2–10. 10.1016/j.ejogrb.2009.02.044 19299064

[91] Wu X Morgan KG Jones CJ Tribe RM Taggart MJ. Myometrial mechanoadaptation during pregnancy: implications for smooth muscle plasticity and remodelling. J Cell Mol Med. (2008) 12:1360–73. 10.1111/j.1582-4934.2008.00306.x 18363833 PMC2729593

[92] Lonard DM O'Malley BW. The expanding cosmos of nuclear receptor coactivators. Cell. (2006) 125:411–4. 10.1016/j.cell.2006.04.021 16678083

[93] Liu D Benlhabib H Mendelson CR. cAMP enhances estrogen-related receptor alpha (ERRalpha) transcriptional activity at the *SP-A* promoter by increasing its interaction with protein kinase A and steroid receptor coactivator 2 (SRC-2). Mol Endocrinol. (2009) 23:772–83. 10.1210/me.2008-0282 19264843 PMC2691680

[94] Yi M Tong GX Murry B Mendelson CR. Role of CBP/p300 and SRC-1 in transcriptional regulation of the pulmonary surfactant protein-A (SP-A) gene by thyroid transcription factor-1 (TTF-1). J Biol Chem. (2001) 277:2997–3005. 10.1074/jbc.M109793200 11713256

[95] Islam KN Mendelson CR. Permissive effects of oxygen on cyclic AMP and interleukin-1 stimulation of surfactant protein A gene expression are mediated by epigenetic mechanisms. Mol Cell Biol. (2006) 26:2901–12. 10.1128/MCB.26.8.2901-2912.2006 16581766 PMC1446958

[96] Xu J Wu RC O'Malley BW. Normal and cancer-related functions of the p160 steroid receptor co-activator (SRC) family. Nat Rev Cancer. (2009) 9:615–30. 10.1038/nrc2695 19701241 PMC2908510

[97] Johnson AB O'Malley BW. Steroid receptor coactivators 1, 2, and 3: critical regulators of nuclear receptor activity and steroid receptor modulator (SRM)-based cancer therapy. Mol Cell Endocrinol. (2012) 348:430–9. 10.1016/j.mce.2011.04.021 21664237 PMC3202666

[98] Mark M Yoshida-Komiya H Gehin M Liao L Tsai MJ O'Malley BW. Partially redundant functions of SRC-1 and TIF2 in postnatal survival and male reproduction. Proc Natl Acad Sci USA. (2004) 101:4453–8. 10.1073/pnas.0400234101 15070739 PMC384768

[99] Hoffman DR Romero R Johnston JM. Detection of platelet-activating factor in amniotic fluid of complicated pregnancies. Am J Obstet Gynecol. (1990) 162:525–8. 10.1016/0002-9378(90)90423-5 2309839

[100] Yasuda K Furukawa M Johnston JM. Effect of estrogens on plasma platelet-activating factor acetylhydrolase and the timing of parturition in the rat. Biol Reprod. (1996) 54:224–9. 10.1095/biolreprod54.1.224 8838020

[101] Zhu YP Hoffman DR Hwang SB Miyaura S Johnston JM. Prolongation of parturition in the pregnant rat following treatment with a platelet activating factor receptor antagonist. Biol Reprod. (1991) 44:39–42. 10.1095/biolreprod44.1.39 1849751

[102] Ishii S Kuwaki T Nagase T Maki K Tashiro F Sunaga S. Impaired anaphylactic responses with intact sensitivity to endotoxin in mice lacking a platelet-activating factor receptor. J Exp Med. (1998) 187:1779–88. 10.1084/jem.187.11.1779 9607919 PMC2212308

[103] Jeanneton O Delvaux M Botella A Frexinos J Bueno L. Platelet-activating factor (PAF) induces a contraction of isolated smooth muscle cells from guinea pig ileum: intracellular pathway involved. J Pharmacol Exp Ther. (1993) 267:31–7. 8229757

[104] Kim BK Ozaki H Lee SM Karaki H. Increased sensitivity of rat myometrium to the contractile effect of platelet activating factor before delivery. Br J Pharmacol. (1995) 115:1211–4. 10.1111/j.1476-5381.1995.tb15027.x 7582547 PMC1908802

[105] Montrucchio G Alloatti G Tetta C Roffinello C Emanuelli G Camussi G. *In vitro* contractile effect of platelet-activating factor on guinea-pig myometrium. Prostaglandins. (1986) 32:539–54. 10.1016/0090-6980(86)90036-5 3797692

[106] Tetta C Montrucchio G Alloatti G Roffinello C Emanuelli G Benedetto C Platelet-activating factor contracts human myometrium *in vitro*. Proc Soc Exp Biol Med. (1986) 83:376–81. 10.3181/00379727-183-42435 3797421

[107] Bridges JP Ikegami M Brilli LL Chen X Mason RJ Shannon JM. LPCAT1 regulates surfactant phospholipid synthesis and is required for transitioning to air breathing in mice. J Clin Invest. (2010) 120:1736–48. 10.1172/JCI38061 20407208 PMC2860922

[108] Chen X Hyatt BA Mucenski ML Mason RJ Shannon JM. Identification and characterization of a lysophosphatidylcholine acyltransferase in alveolar type II cells. Proc Natl Acad Sci USA. (2006) 103:11724–9. 10.1073/pnas.0604946103 16864775 PMC1544237

[109] Nakanishi H Shindou H Hishikawa D Harayama T Ogasawara R Suwabe A. Cloning and characterization of mouse lung-type acyl-CoA:lysophosphatidylcholine acyltransferase 1 (LPCAT1). Expression in alveolar type II cells and possible involvement in surfactant production. J Biol Chem. (2006) 281:20140–7. 10.1074/jbc.M600225200 16704971

[110] Hardy DB Janowski BA Corey DR Mendelson CR Progesterone receptor (PR) plays a major anti-inflammatory role in human myometrial cells by antagonism of NF-kappaB activation of cyclooxygenase 2 (COX-2) expression. Mol Endocrinol. (2006) 20:2724–33. 10.1210/me.2006-0112 16772530

[111] Dong X Yu C Shynlova O Challis JR Rennie PS Lye SJ. p54nrb is a transcriptional corepressor of the progesterone receptor that modulates transcription of the labor-associated gene, connexin 43 (Gja1). Mol Endocrinol. (2009) 23:1147–60. 10.1210/me.2008-0357 19423654 PMC5419194

[112] Dong X Shylnova O Challis JR Lye SJ. Identification and characterization of the protein-associated splicing factor as a negative co-regulator of the progesterone receptor. J Biol Chem. (2005) 280:13329–40. 10.1074/jbc.M409187200 15668243

[113] Deroo BJ Archer TK. Differential activation of the IκBα and mouse mammary tumor virus promoters by progesterone and glucocorticoid receptors. J Steroid Biochem Mol Biol. (2002) 81:309–17. 10.1016/S0960-0760(02)00072-9 12361720

[114] Chen CC Hardy DB Mendelson CR. Progesterone receptor inhibits proliferation of human breast cancer cells via induction of MAPK phosphatase 1 (MKP-1/DUSP1). J Biol Chem. (2011) 286:43091–102. 10.1074/jbc.M111.295865 22020934 PMC3234857

[115] Vicent GP Ballare C Nacht AS Clausell J Subtil-Rodriguez A Quiles I. Induction of progesterone target genes requires activation of Erk and Msk kinases and phosphorylation of histone H3. Mol Cell. (2006) 24:367–81. 10.1016/j.molcel.2006.10.011 17081988

[116] Spoelstra NS Manning NG Higashi Y Darling D Singh M Shroyer KR. The transcription factor ZEB1 is aberrantly expressed in aggressive uterine cancers. Cancer Res. (2006) 66:3893–902. 10.1158/0008-5472.CAN-05-2881 16585218

[117] Hardy DB Mendelson CR Progesterone receptor (PR) antagonism of the inflammatory signals leading to labor. Fetal Maternal Med Rev. (2006) 17:281–9. 10.1017/S0965539506001811

[118] Siiteri PK Stites DP. Immunologic and endocrine interrelationships in pregnancy. Biol Reprod. (1982) 26:1–14. 10.1095/biolreprod26.1.1 7039702

[119] Tan H Yi L Rote NS Hurd WW Mesiano S. Progesterone receptor-A and -B have opposite effects on proinflammatory gene expression in human myometrial cells: implications for progesterone actions in human pregnancy and parturition. J Clin Endocrinol Metab. (2012) 97:E719–30. 10.1210/jc.2011-3251 22419721 PMC3339884

[120] Shynlova O Lee YH Srikhajon K Lye SJ. Physiologic uterine inflammation and labor onset: integration of endocrine and mechanical signals. Reprod Sci. (2013) 20:154–67. 10.1177/1933719112446084 22614625

[121] Havelock JC Keller P Muleba N Mayhew BA Casey BM Rainey WE. Human myometrial gene expression before and during parturition. Biol Reprod. (2005) 72:707–19. 10.1095/biolreprod.104.032979 15509731

[122] Kalkhoven E Wissink S van der Saag PT van der BB. Negative interaction between the RelA(p65) subunit of NF-κB and the progesterone receptor. J Biol Chem. (1996) 271:6217–24. 10.1074/jbc.271.11.6217 8626413

[123] Baldwin ASJ The NF-kappaB and IkappaB proteins: new discoveries and insights. Annu Rev Immunol. (1996) 14:649–83. 10.1146/annurev.immunol.14.1.649 8717528

[124] Miller L Hunt JS. Regulation of TNF-alpha production in activated mouse macrophages by progesterone. J Immunol. (1998) 160:5098–104. 9590261

[125] Hardy DB Janowski BA Chen CC Mendelson CR. Progesterone receptor inhibits aromatase and inflammatory response pathways in breast cancer cells via ligand-dependent and ligand-independent mechanisms. Mol Endocrinol. (2008) 22:1812–24. 10.1210/me.2007-0443 18483177 PMC2725768

[126] Vanden Berghe W Plaisance S Boone E De Bosscher K Schmitz ML Fiers W. p38 and extracellular signal-regulated kinase mitogen-activated protein kinase pathways are required for nuclear factor-κB p65 transactivation mediated by tumor necrosis factor. J Biol Chem. (1998) 273:3285–90. 10.1074/jbc.273.6.3285 9452444

[127] Gupta SC Sundaram C Reuter S Aggarwal BB. Inhibiting NF-κB activation by small molecules as a therapeutic strategy. Biochim Biophys Acta. (2010) 1799:775–87. 10.1016/j.bbagrm.2010.05.004 20493977 PMC2955987

[128] Kyriakis JM. Activation of the AP-1 transcription factor by inflammatory cytokines of the TNF family. Gene Expr. (1999) 7:217–31. 10440223 PMC6174675

[129] Mitchell JA Lye SJ. Differential activation of the connexin 43 promoter by dimers of activator protein-1 transcription factors in myometrial cells. Endocrinology. (2005) 146:2048–54. 10.1210/en.2004-1066 15618352

[130] Nadeem L Shynlova O Matysiak-Zablocki E Mesiano S Dong X Lye S. Molecular evidence of functional progesterone withdrawal in human myometrium. Nat Commun. (2016) 7:11565. 10.1038/ncomms11565 27220952 PMC4894948

[131] Williams KC Renthal NE Condon JC Gerard RD Mendelson CR. MicroRNA-200a serves a key role in the decline of progesterone receptor function leading to term and preterm labor. Proc Natl Acad Sci USA. (2012) 109:7529–34. 10.1073/pnas.1200650109 22529366 PMC3358858

[132] Peters GA Yi L Skomorovska-Prokvolit Y Patel B Amini P Tan H Inflammatory stimuli increase progesterone receptor-A stability and transrepressive activity in myometrial cells. Endocrinology. (2016) 58:en2016–1537. 10.1210/en.2016-1537 PMC5412979 27886516

[133] Amini P Michniuk D Kuo K Yi L Skomorovska-Prokvolit Y Peters GA. Human parturition involves phosphorylation of progesterone receptor-A at serine-345 in myometrial cells. Endocrinology. (2016) 157:4434–45. 10.1210/en.2016-1654 27653036 PMC5086536

[134] Cochrane DR Spoelstra NS Richer JK. The role of miRNAs in progesterone action. Mol Cell Endocrinol. (2012) 357:50–9. 10.1016/j.mce.2011.09.022 21952083

[135] Brabletz S Bajdak K Meidhof S Burk U Niedermann G Firat E. The ZEB1/miR-200 feedback loop controls Notch signalling in cancer cells. EMBO J. (2011) 30:770–82. 10.1038/emboj.2010.349 21224848 PMC3041948

[136] Bracken CP Gregory PA Kolesnikoff N Bert AG Wang J Shannon MF. A double-negative feedback loop between ZEB1-SIP1 and the microRNA-200 family regulates epithelial-mesenchymal transition. Cancer Res. (2008) 68:7846–54. 10.1158/0008-5472.CAN-08-1942 18829540

[137] Burk U Schubert J Wellner U Schmalhofer O Vincan E Spaderna S. A reciprocal repression between ZEB1 and members of the miR-200 family promotes EMT and invasion in cancer cells. EMBO Rep. (2008) 9:582–9. 10.1038/embor.2008.74 18483486 PMC2396950

[138] Gregory PA Bert AG Paterson EL Barry SC Tsykin A Farshid G et a. The miR-200 family and miR-205 regulate epithelial to mesenchymal transition by targeting ZEB1 and SIP1. Nat Cell Biol. (2008) 10:593–601. 10.1038/ncb1722 18376396

[139] Loebel DA Tsoi B Wong N Tam PP. A conserved noncoding intronic transcript at the mouse Dnm3 locus. Genomics. (2005) 85:782–9. 10.1016/j.ygeno.2005.02.001 15885504

[140] Chakrabarty A Tranguch S Daikoku T Jensen K Furneaux H Dey SK. MicroRNA regulation of cyclooxygenase-2 during embryo implantation. Proc Natl Acad Sci USA. (2007) 104:15144–9. 10.1073/pnas.0705917104 17848513 PMC1986627

[141] Puri CP Garfield RE. Changes in hormone levels and gap junctions in the rat uterus during pregnancy and parturition. Biol Reprod. (1982) 27:967–75. 10.1095/biolreprod27.4.967 6959654

[142] Power SG Challis JR. The effects of gestational age and intrafetal ACTH administration on the concentration of progesterone in the fetal membranes, endometrium, and myometrium of pregnant sheep. Can J Physiol Pharmacol. (1987) 65:136–40. 10.1139/y87-027 3032381

[143] Csapo AI Eskola J Tarro S. Gestational changes in the progesterone and prostaglandin F levels of the guinea-pig. Prostaglandins. (1981) 21:53–64. 10.1016/0090-6980(81)90196-9 7208954

[144] Runnebaum B Zander J Progesterone and 20alpha-dihydroprogesterone in human myometrium during pregnancy. Acta Endocrinol Suppl. (1971) 150:3–45. 10.1530/acta.0.066S005 5279327

[145] Richer JK Lange CA Manning NG Owen G Powell R Horwitz KB. Convergence of progesterone with growth factor and cytokine signaling in breast cancer. Progesterone receptors regulate signal transducers and activators of transcription expression and activity. J Biol Chem. (1998) 273:31317–26. 10.1074/jbc.273.47.31317 9813040

[146] Shynlova O Oldenhof A Dorogin A Xu Q Mu J Nashman N. Myometrial apoptosis: activation of the caspase cascade in the pregnant rat myometrium at midgestation. Biol Reprod. (2006) 74:839–49. 10.1095/biolreprod.105.048124 16407500

[147] Jeyasuria P Wetzel J Bradley M Subedi K Condon JC. Progesterone-regulated caspase 3 action in the mouse may play a role in uterine quiescence during pregnancy through fragmentation of uterine myocyte contractile proteins. Biol Reprod. (2009) 80:928–34. 10.1095/biolreprod.108.070425 19144964 PMC6354714

[148] Kyathanahalli C Organ K Moreci RS Anamthathmakula P Hassan SS Caritis SN. Uterine endoplasmic reticulum stress-unfolded protein response regulation of gestational length is caspase-3 and−7-dependent. Proc Natl Acad Sci USA. (2015) 112:14090–5. 10.1073/pnas.1518309112 26504199 PMC4653163

[149] Suresh A Subedi K Kyathanahalli C Jeyasuria P Condon JC. Uterine endoplasmic reticulum stress and its unfolded protein response may regulate caspase 3 activation in the pregnant mouse uterus. PLoS ONE. (2013) 8:e75152. 10.1371/journal.pone.0075152 24058658 PMC3772854

[150] Wei LL Norris BM Baker CJ. An N-terminally truncated third progesterone receptor protein, PR(C), forms heterodimers with PR(B) but interferes in PR(B)-DNA binding. J Steroid Biochem Mol Biol. (1997) 62:287–97. 10.1016/S0960-0760(97)00044-7 9408082

[151] Wei LL Gonzalez-Aller C Wood WM Miller LA Horwitz KB. 5'-Heterogeneity in human progesterone receptor transcripts predicts a new amino-terminal truncated “C”-receptor and unique A-receptor messages. Mol Endocrinol. (1990) 4:1833–40. 10.1210/mend-4-12-1833 2082185

[152] Wei LL Hawkins P Baker C Norris B Sheridan PL Quinn PG. An amino-terminal truncated progesterone receptor isoform, PRc, enhances progestin-induced transcriptional activity. Mol Endocrinol. (1996) 10:1379–87. 10.1210/me.10.11.1379 8923464

[153] Samalecos A Gellersen B. Systematic expression analysis and antibody screening do not support the existence of naturally occurring progesterone receptor (PR)-C, PR-M, or other truncated PR isoforms. Endocrinology. (2008) 149:5872–87. 10.1210/en.2008-0602 18617611

[154] Li X Wong J Tsai SY Tsai MJ O'Malley BW. Progesterone and glucocorticoid receptors recruit distinct coactivator complexes and promote distinct patterns of local chromatin modification. Mol Cell Biol. (2003) 23:3763–73. 10.1128/MCB.23.11.3763-3773.2003 12748280 PMC155204

[155] Mukherjee A Soyal SM Fernandez-Valdivia R Gehin M Chambon P DeMayo FJ. Steroid receptor coactivator 2 is critical for progesterone-dependent uterine function and mammary morphogenesis in the mouse. Mol Cell Biol. (2006) 26:6571–83. 10.1128/MCB.00654-06 16914740 PMC1592830

[156] Condon JC Jeyasuria P Faust JM Wilson JM Mendelson CR A decline in progesterone receptor coactivators in the pregnant uterus at term may antagonize progesterone receptor function and contribute to the initiation of labor. Proc Natl Acad Sci USA. (2003) 100:9518–23. 10.1073/pnas.1633616100 12886011 PMC170950

[157] Lindstrom TM Mohan AR Johnson MR Bennett PR. Histone deacetylase inhibitors exert time-dependent effects on nuclear factor-κB but consistently suppress the expression of proinflammatory genes in human myometrial cells. Mol Pharmacol. (2008) 74:109–21. 10.1124/mol.107.042838 18375836

[158] Moynihan AT Hehir MP Sharkey AM Robson SC Europe-Finner GN Morrison JJ. Histone deacetylase inhibitors and a functional potent inhibitory effect on human uterine contractility. Am J Obstet Gynecol. (2008) 199:167. 10.1016/j.ajog.2008.01.002 18455134

[159] Leite RS Brown AG Strauss JF III. Tumor necrosis factor-α suppresses the expression of steroid receptor coactivator-1 and−2: a possible mechanism contributing to changes in steroid hormone responsiveness. FASEB J. (2004) 18:1418–20. 10.1096/fj.04-1684fje 15231721

[160] Mendelson CR. Minireview: fetal-maternal hormonal signaling in pregnancy and labor. Mol Endocrinol. (2009) 23:947–54. 10.1210/me.2009-0016 19282364 PMC2703595

[161] Ishida M Choi JH Hirabayashi K Matsuwaki T Suzuki M Yamanouchi K. Reproductive phenotypes in mice with targeted disruption of the 20alpha-hydroxysteroid dehydrogenase gene. J Reprod Dev. (2007) 53:499–508. 10.1262/jrd.18125 17272929

[162] Haraguchi H Saito-Fujita T Hirota Y Egashira M Matsumoto L Matsuo M. MicroRNA-200a locally attenuates progesterone signaling in the cervix, preventing embryo implantation. Mol Endocrinol. (2014) 28:1108–17. 10.1210/me.2014-1097 24850415 PMC4075165

